# Tannic Acid–Iron Complex-Based Nanoparticles as a Novel Tool against Oxidative Stress

**DOI:** 10.1021/acsami.1c24576

**Published:** 2022-03-30

**Authors:** Carlotta Pucci, Chiara Martinelli, Daniele De Pasquale, Matteo Battaglini, Nicoletta di Leo, Andrea Degl’Innocenti, Melike Belenli Gümüş, Filippo Drago, Gianni Ciofani

**Affiliations:** † Smart Bio-Interfaces, Istituto Italiano di Tecnologia, Viale Rinaldo Piaggio 34, 56025 Pontedera, Italy; ‡ The Biorobotics Institute, Scuola Superiore Sant’Anna, Viale Rinaldo Piaggio 34, 56025 Pontedera, Italy; § Electron Microscopy Facility, Istituto Italiano di Tecnologia, Via Morego 30, 16163 Genova, Italy

**Keywords:** tannic acid−iron complexes, oxidative stress, antioxidant, bionanomaterials, planarians

## Abstract

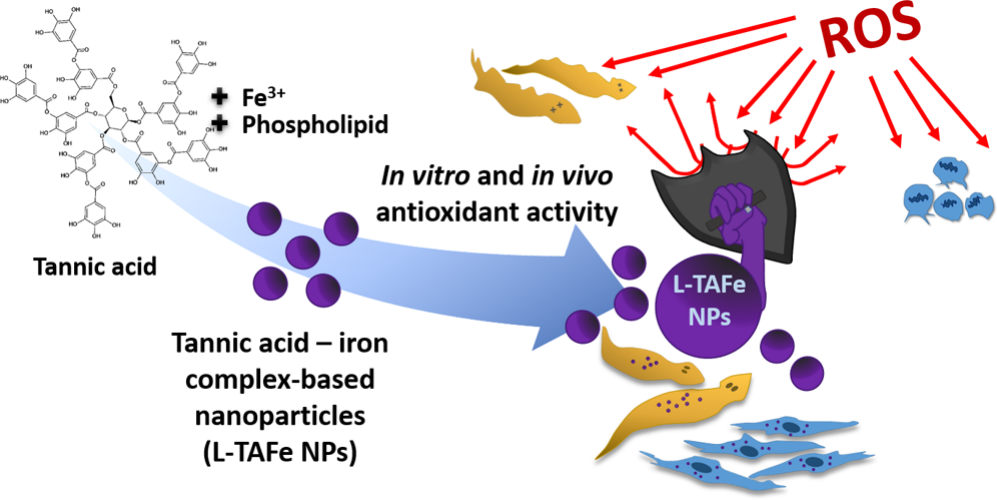

Accumulation of reactive oxygen species in cells leads to oxidative stress, with consequent damage for cellular components and activation of cell-death mechanisms. Oxidative stress is often associated with age-related conditions, as well as with several neurodegenerative diseases. For this reason, antioxidant molecules have attracted a lot of attention, especially those derived from natural sources—like polyphenols and tannins. The main issue related to the use of antioxidants is their inherent tendency to be oxidized, their quick enzymatic degradation in biological fluids, and their poor bioavailability. Nanomedicine, in this sense, has helped in finding new solutions to deliver and protect antioxidants; however, the concentration of the encapsulated molecule in conventional nanosystems could be very low and, therefore, less effective. We propose to exploit the properties of tannic acid, a known plant-derived antioxidant, to chelate iron ions, forming hydrophobic complexes that can be coated with a biocompatible and biodegradable phospholipid to improve stability in biological media. By combining nanoprecipitation and hot sonication procedures, we obtained three-dimensional networks composed of tannic acid–iron with a hydrodynamic diameter of ≈200 nm. These nanostructures show antioxidant properties and scavenging activity in cells after induction of an acute chemical pro-oxidant insult; moreover, they also demonstrated to counteract damage induced by oxidative stress both *in vitro* and on an *in vivo* model organism (planarians).

## Introduction

In healthy cells, the equilibrium between the production of reactive oxygen species (ROS) as byproducts of our physiological metabolism and their removal by intracellular enzymes is maintained in a precise balance to guarantee correct cell signaling and function.^1,2^ However, if ROS are not properly scavenged, their excessive accumulation results in oxidative stress that leads to severe damage to cellular components (e.g., lipids, proteins, nucleic acids) and/or to the activation of cell-death mechanisms.^3,4^ Oxidative stress has been linked to many age-related conditions and neurodegenerative diseases, such as Alzheimer’s and Parkinson’s diseases.^2^

In recent years, organic antioxidants have been recognized as potential therapeutic agents for several oxidative stress-associated disorders. In particular, phytochemicals have been studied for their beneficial properties to human health.^5^ Although administration of antioxidant supplements in the daily diet has demonstrated to be effective in treating diseases induced by oxidative stress, their clinical application remains limited due to their poor solubility in water, low bioavailability, and susceptibility to degradation.^6^ Advances in nanomedicine brought innovative tools for antioxidant delivery;^2,7^ for example, curcumin, a water-insoluble phytochemical antioxidant, has been effectively loaded in cubosomes, alone or in combination with catalase, and the antioxidant activity in the nanotechnological system was demonstrated to be higher than that one of free curcumin.^8,9^ The encapsulation of polyphenols—natural antioxidants present in vegetables and influencing many cellular pathways^10−12^—into *ad hoc* nanocarriers has demonstrated to protect them from oxidation.^13,14^ Indeed, nanoparticles can be loaded with effective amounts of molecules, increasing stability and pharmacokinetic properties.^15^ Several kinds of nanocarriers have been developed and are currently under evaluation in preclinical and clinical trials.

Tannic acid is a high-molecular-weight, plant-derived polyphenol belonging to the family of tannins, and it is usually present in wine, coffee, tea, as well as in some fruits such as grape and banana.^16^ Tannic acid is a decagalloyl residue with a center glucose molecule esterified at all its hydroxyl groups with 10 gallic acid units (Figure S1). It possesses strong antioxidant properties^16,17^ but, being soluble in water, it is difficult to be efficiently encapsulated in nanoparticles, protecting it from degradation. Notably, tannic acid has been covalently attached to poly(metacrylic acid) to obtain tannic acid-decorated nanoparticles, showing an improved antioxidant activity.^18^ Moreover, in its free form, tannic acid can interact with plasma proteins, causing their precipitation,^19^ and it can act as a chelating agent for metal ions naturally present in our body.^20^ On the other hand, such chelating abilities can be advantageous to prepare more hydrophobic tannic acid–metal complexes with desired properties.^21,22^ Fe^3+^ is usually the preferred metal ion to coordinate tannic acid, thanks to its strong interaction with the ligand, high linkability, and low toxicity as compared to other metal ions that are known to also induce tannic acid cross-linking.^22,23^ Depending on the pH and concentration, tannic acid–Fe^3+^ complexes have very poor solubility in water;^21^ thus, they can be easily encapsulated or coated with amphiphilic compounds that can enhance their bioavailability while protecting tannic acid from degradation. Tannic acid–Fe^3+^ or, more in general, tannic acid–metal ion complexes have been proposed for several biological applications, such as cancer therapy, imaging, or wound healing.^22^ However, to the best of our knowledge, only Tang et al. considered the use of tannic acid–Fe^3+^ complexes to exploit the antioxidant properties of tannic acid in biological systems.^24^ Moreover, the authors proposed a flash nanoprecitation strategy in which tannic acid–Fe^3+^ complexes were formed in the presence of an amphiphilic block copolymer (polystyrene-*b*-poly(ethylene glycol)) to form nanoparticles, rather than thin films or capsules previously put forth by other researchers. In our work, we synthetized nanoparticles made by tannic acid–Fe^3+^ complexes stabilized with a PEGylated phospholipid widely used in biomedical applications for its biocompatibility and biodegradability, that is 1,2-distearoyl-*sn*-glycero-3-phosphoethanolamine-poly(ethylene glycol) (DSPE-PEG).^25^ Moreover, the presence of PEG is known to impart steric stability and prolonged circulation time to nanoparticles, while preventing rapid clearance by the mononuclear phagocytic system.^26^ Different from Tang et al.,^24^ DSPE-PEG-coated tannic acid–Fe^3+^ nanoparticles (from now on referred to as L-TAFe NPs) have been obtained by a two-step formulation procedure, combining nanoprecipitation (for the complex formation) and hot sonication (for the final coating with DSPE-PEG). The coating guarantees a better protection of the tannic acid–Fe^3+^ complexes from the external environment and should allow an easy further functionalization for future different applications.

In this work, we also provide a detailed characterization of L-TAFe NPs, focusing on their morphology, physicochemical properties, and stability in several conditions, giving useful information on the relative content of each component of the complex L-TAFe NPs system. Their scavenging activity toward different kinds of radicals has been tested with several assays and compared to the corresponding free tannic acid and to other usual antioxidant compounds (l-ascorbic acid^27,28^ and N-acetyl-l-cysteine^29,30^). While the antioxidant properties of these molecular antioxidants have been already characterized in the past, a study on L-TAFe NPs is still missing and could give important cues to researchers approaching this antioxidant system. A more comprehensive study on their biocompatibility, interaction with cells, antioxidant activity, and protective effects against damage induced by oxidative stress *in vitro* on human primary skin fibroblasts has been conducted to demonstrate the potentialities of this system with respect to bare molecular antioxidants. Finally, L-TAFe NPs have also been tested *in vivo* on planarians, free-living freshwater flatworms (phylum Platyhelminthes). Planarians are widely used in toxicological assays,^31−33^ and they have already been experimental subjects for nanomaterial studies.^34,35^ These organisms are not only utilized for their excellent regeneration capabilities, yet also because they share some fundamental features with more complex vertebrates, such as the overall body plan or the neuronal structure and activity.^36^ A further advantage of deploying planarians in toxicological studies is represented by their low cost and easy handling with respect to other model animals.^31^ In this work, L-TAFe NPs were tested for antioxidant activity in the planarian species *Dugesia japonica*, particularly suitable for high-throughput toxicological screenings.^37^

## Materials and Methods

### Synthesis of L-TAFe NPs

L-TAFe NPs were fabricated by mixing 2 mL of a solution of 4 mg/mL tannic acid (Sigma-Aldrich) and 2 mg/mL DSPE-PEG (5000 Da, Nanocs, Inc.) in dimethyl sulfoxide with 2 mL of 2 mg/mL FeCl_3_ (Sigma-Aldrich) in MilliQ water under stirring. The resulting solution turned immediately purple as tannic acid–iron complexes started to form. To quench the formation of network complexes, 4 mL of MilliQ water was added. The final dispersion is unstable due to the hydrophobicity of the complexes: therefore, 10 mg of DSPE-PEG was added to the dispersion to impart stability, and the mixture was sonicated for 10 min (amplitude 90%) using an ultrasonic tip (Fisherbrand Q125 Sonicator), giving rise to a stable purple solution. Finally, L-TAFe NPs were purified by centrifugation with Amicon filters (Ultra-4 Centrifugal Filter Unit [MWCO 100 kDa], Sigma-Aldrich) at 2460*g* for 15 min at 15 °C three times and finally redispersed in MilliQ water. The concentration of the samples (in mg/mL) was determined by weighting the dehydrated nanoparticles after freeze-drying known aliquots of the dispersion.

### Physicochemical Characterization

Transmission electron microscopy (TEM) was performed to obtain information about the size and morphology of the nanoparticles. A drop of the sample was deposited on a Cu grid (150 mesh) coated with an ultrathin amorphous carbon film pretreated with plasma to clean the surface. The drop was removed with a filter paper. Images were acquired with a JEM-1011 transmission electron microscope (JEOL) at 100 kV on a single-tilt sample holder.

Dynamic light scattering and ζ-potential measurements were carried out using a NanoZS90 Zeta-sizer (Malvern Instruments Ltd.) to evaluate the hydrodynamic diameter and the ζ-potential of L-TAFe NPs. Measurements were performed on 100 μg/mL L-TAFe NPs in MilliQ water at 37 °C. The stability of L-TAFe NPs in water, phosphate-buffered saline solution (PBS, Sigma-Aldrich), Dulbecco’s modified Eagle’s medium (DMEM, Sigma-Aldrich) with 10% fetal bovine serum (FBS, Sigma-Aldrich), and DMEM + FBS (10%) + H_2_O_2_ (100 μM, to simulate oxidative stress conditions) was evaluated by diluting a nanoparticle stock solution (4 mg/mL) in the corresponding buffer up to a final concentration of 100 μg/mL. The intensity distribution was derived from the correlogram through CONTIN analysis, whereas the hydrodynamic diameter and the polydispersity index was obtained from cumulant analysis.

UV/vis spectroscopy was carried out with a Lambda 45 UV/vis spectrometer (PerkinElmer) in the range 350–800 nm at room temperature to highlight the formation of the tannic acid–iron complexes due to the formation of a ligand-to-metal charge transfer band.

Fourier-transform infrared (FTIR) spectroscopy was carried out with a Miracle 10 (Shimadzu) on freeze-dried samples in the range 500–4000 cm^–1^, performing 16 scans at a resolution step of 4 cm^–1^.

Raman spectroscopy (LabRAM HR Evolution, Horiba) was performed to detect functional groups and, in particular, the formation of Fe–O bonding as a consequence of L-TAFe NP formation. Spectra were acquired with a 532 nm laser in the range of 200–2000 cm^–1^.

Thermogravimetric analysis (TGA) of freeze-dried samples was performed using a TGA Q50 (TA Instruments), increasing the temperature from 30 to 700 °C at a heating rate of 5 °C/min and under a nitrogen flow (50 mL/min). L-TAFe NP thermogram, and in particular its weight derivative (%/°C), has been analyzed with Originpro 9.1, using the Multipeak Fit tool and setting the shape of the peaks as Gaussian.

To quantify the Fe content in L-TAFe NPs, an elemental analysis *via* inductively coupled plasma optical emission spectroscopy (ICP-OES) was performed, with an iCAP-7600 DUO (Thermo Fisher Scientific). Eleven milligrams of freeze-dried L-TAFe NPs were digested in 1 mL of a mixture of 2:1 nitric acid (HNO_3_ 60% v/v) and hydrogen peroxide (H_2_O_2_ ≥30%). The blend was left under sonication at 65 °C for 2 h. Samples were diluted using ultrapure water to reach a final volume of 10 mL. Before measurement, first, the solution was filtered with a 0.45 μm regenerated cellulose filter and then with a 0.45-μm poly(tetrafluoroethylene) (PTFE) filter. Measurements have been carried out using a plasma power of 1150 W, a nebulizer gas flow of 0.5 L/min, a cool flow of 12 L/min, and an aux flow of 0.5 L/min. The Fe amount was detected at 238.20 nm.

### Antioxidant Activity Evaluation

The antioxidant activity of L-TAFe NPs was evaluated by measuring the total non-enzymatic antioxidant capacity (TAC). This assay is based on the reduction of Cu^2+^ ions to Cu^+^ ions. The reduced Cu^+^ ion chelates with a colorimetric probe and, as a consequence, an absorbance peak, proportional to the TAC, can be detected at ∼570 nm. The antioxidant capacity is then converted in Trolox equivalents. Trolox is a water-soluble analogue of vitamin E used as an antioxidant standard. Trolox standards and samples were prepared according to the manufacturer’s instructions (MAK187, Sigma-Aldrich). Three concentrations of L-TAFe NPs were assayed (10, 50, 100 μg/mL) to ensure that the assay was being performed in the linearity range (Figure S2). Upon assay reaction incubation, absorbance was measured at 570 nm. A Trolox standard curve was plotted, and Trolox equivalents of the samples were determined from it.

The total radical scavenging activity was evaluated on three different concentrations of L-TAFe NPs, namely, 10, 50, and 100 μg/mL, with the α,α-diphenyl-β-picrylhydrazyl (DPPH) assay.^38^ Two hundred microliters of a 0.35 mM stock solution of DPPH (Sigma-Aldrich) in methanol was added to a mixture of 500 μL of pure methanol and 1050 μL of a dispersion of L-TAFe NPs at the desired concentration in water. In each sample, the concentration of DPPH was equal to 50 μM. DPPH absorbance at 526 nm was measured (Lambda 45 UV/vis spectrometer, PerkinElmer) at different time points (*t* = 0, 10, 20, 30, 60 min) from the addition of DPPH to L-TAFe NP dispersions. Control samples with DPPH alone and tannic acid, l-ascorbic acid, and N-acetyl-l-cysteine (5 μM, that is, the estimated concentration of tannic acid in L-TAFe NPs 10 μg/mL) were also assessed. The scavenging activity (%) of L-TAFe NPs at each time point was calculated using eq 1
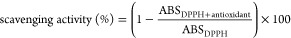
1where ABS_DPPH + antioxidant_ represents the absorbance at 526 nm of DPPH in the presence of L-TAFe NPs at the selected time point, while ABS_DPPH_ represents the absorbance at 526 nm of DPPH alone at the same time point.

Hydrogen peroxide (H_2_O_2_) scavenging activity was evaluated by adapting a previously reported method.^16^ Briefly, 2 μL of H_2_O_2_ (30%, Sigma-Aldrich) was added to 1998 μL of a solution of the antioxidant to be tested in phosphate buffer (0.1 M, pH 7.4). The samples chosen for this experiment were L-TAFe NPs (10 μg/mL), tannic acid, l-ascorbic acid, and N-acetyl-l-cysteine (5 μM). H_2_O_2_ absorbance at 230 nm was measured (Lambda 45 UV/vis spectrometer, PerkinElmer) in a quartz cuvette. All of the samples were normalized with respect to a blank solution (either with or without antioxidant) without H_2_O_2_. The percentage of H_2_O_2_ scavenging (%) was evaluated using eq 2
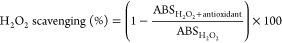
2where ABS_H_2_O_2_ + antioxidant_ represents the absorbance at 230 nm of H_2_O_2_ in the presence of any antioxidant tested, while ABS_H_2_O_2__ represents the absorbance at 230 nm of H_2_O_2_ alone.

The scavenging activity against singlet oxygen was evaluated by monitoring the bleaching of 1,3-diphenylisobenzofuran (DPBF, 50 μM, Sigma-Aldrich) in the presence of singlet oxygen produced by the reaction between NaOCl (50 mM) and H_2_O_2_ (50 mM) in 45 mM phosphate buffer (pH 7.1) and in the presence of 50 mM histidine and antioxidants tested (L-TAFe NPs (10 μg/mL), tannic acid, l-ascorbic acid, and N-acetyl-l-cysteine (5 μM)).^39,40^ The mixture was incubated at 30 °C for 40 min and the absorbance of DPBF measured at 450 nm. Due to the interaction with singlet oxygen, the π-system of DPBF is broken, with consequent bleaching of the molecule; therefore, the reduction in absorbance is correlated to the amount of singlet oxygen in the solution. The DPBF absorbance reduction was determined through eq 3
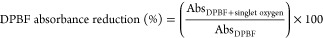
3where Abs_DBPF + singlet oxygen_ is the absorbance at 450 nm in the presence of NaOCl and H_2_O_2_ (with or without the antioxidants) and Abs_DBPF_ is the absorbance at 450 nm without NaOCl and H_2_O_2_ (with or without the antioxidants). Samples without NaOCl and H_2_O_2_ were also incubated at 30 °C for 40 min.

### *In Vitro* Tests

Human primary skin fibroblasts derived from skin punch biopsies were cultured in DMEM high glucose (Sigma-Aldrich) supplemented with 10% heat-inactivated FBS (Sigma-Aldrich), 2 mM l-glutamine (Gibco), 1 mM sodium pyruvate (Gibco), 100 IU/mL penicillin, and 100 μg/mL streptomycin (Gibco). Human fibroblasts were collected with informed consent according to standard procedures for diagnostic skin biopsies and treated according to the standards of good clinical practice.

For biocompatibility evaluation, cells were seeded in 24-well cell culture plates (COSTAR) at a density of 1.5 × 10^4^ cells/well. After 24 h, the cells were incubated with L-TAFe NPs at different concentrations (100, 300, 500, 1000 μg/mL) in complete DMEM without phenol red (Sigma-Aldrich). Water-soluble tetrazolium 1 (WST-1) assay was performed at 24 and 72 h after incubation at 37 °C, 5% CO_2_. The cell proliferation reagent WST-1 (Roche) was diluted (1:20) in 300 μL/well of DMEM without phenol red, and the plates were incubated for 30 min at 37 °C, 5% CO_2_. The absorbance reading was performed with a Victor X3 multiplate reader (PerkinElmer), setting absorbance wavelength at 450 nm and 0.1 s measurement time. Finally, the cells were rinsed with PBS and stored at −80 °C. Frozen samples were successively treated with Quant-iT PicoGreen dsDNA Assay Kit (Invitrogen) to assess the proliferation rate. Samples stocked at −80 °C were subjected to three cycles of freeze/thaw allowing cell lysis and DNA release. The reagent and buffer were diluted according to the manufacturer’s instructions. Fluorescence was measured with a Victor X3 multiplate reader (PerkinElmer), setting the excitation wavelength at 485 nm, emission wavelength at 535 nm, and 0.1 s measurement time. Absorbance (for WST-1) and fluorescence (for PicoGreen) of each experimental class were normalized with respect to untreated controls and expressed as percentage.

The metabolic rate of human primary skin fibroblasts treated with L-TAFe NPs was evaluated with the Alamar Blue assay (Invitrogen). The cells were seeded in 24-well cell culture plates (COSTAR) at a density of 1.5 × 10^4^ cells/well and incubated, after 24 h, with L-TAFe NPs at different concentrations (100, 300, 500, 1000 μg/mL) for 24 and 72 h. Successively, the cells were washed in PBS and incubated with Alamar Blue (1:10 dilution in DMEM without phenol red) for 2 h at 37 °C. The supernatant was collected and the absorbance at both 570 nm (λ_1_) and 600 nm (λ_2_) was measured with a Victor X3 multiplate reader (PerkinElmer). The metabolic rate (with respect to untreated cells) was calculated using eq 4
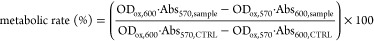
4where OD_ox,600_ and OD_ox,570_ are the molar extinction coefficients of the oxidized form of Alamar Blue at 600 and 570 nm (80 586 and 117 216 M^–1^ cm^–1^, respectively),^41^ while Abs_570,sample_, Abs_600,sample_, Abs_570,CTRL_, and Abs_600,CTRL_ are, respectively, the absorbance values measured at 570 and 600 nm for both the sample and the control cells.

The Trypan Blue exclusion assay was performed to evaluate the percentage of viable cells after treatment with different concentrations of L-TAFe NPs. Human primary skin fibroblasts were seeded in 24-well cell culture plates (COSTAR) at a density of 1.5 × 10^4^ cells/well; after 24 h, they were incubated with L-TAFe NPs at different concentrations (0, 100, 300, 500, 1000 μg/mL) for 24 and 72 h. After the treatment, the cells were washed with PBS, collected, and centrifuged at 2600 rpm for 6 min. The pellet was resuspended in 100 μL of PBS, and 100 μL of a solution of 0.4% Trypan Blue (Sigma-Aldrich) was added. The mixture was incubated for 2 min at room temperature. Afterward, a drop of the treated cell suspension was loaded on a hemacytometer and both unstained (viable) and stained (nonviable) cells were counted. The percentage of viable cells was calculated using eq 5
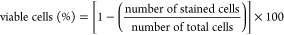
5

For internalization studies, L-TAFe NPs were labeled with Vybrant 3,3′-dioctadecyloxacarbocyanine perchlorate (DiO) cell-labeling solution (Thermo Fisher) by incubation with 5 μM dye for 2 h at 37 °C. After performing three washes with Amicon Ultra-4 Centrifugal Filter Units (MWCO 100 kDa, Sigma-Aldrich), the final pellet was resuspended in sterile MilliQ water. Human primary skin fibroblasts were seeded in 24-well black μ-plate IbiTreat (Ibidi) at a density of 2 × 10^4^ cells/well. After 24 h, they were incubated with 100 μg/mL of Vybrant DiO-labeled L-TAFe NPs in complete DMEM without phenol red (Sigma-Aldrich) at 37 °C, 5% CO_2_. After 24 and 72 h of incubation, fibroblasts were rinsed with PBS and fixed with 4% paraformaldehyde at 4 °C for 30 min. Staining was performed by incubating cells with Hoechst 33342 dye (1 μg/mL, Thermo Scientific) and tetramethylrhodamine-phalloidin (2.5 μg/mL, Sigma-Aldrich) in 10% goat serum (Euroclone) for 90 min at 37 °C. After a final rinse with PBS, the plates were stored at 4 °C in the dark. A C2s confocal laser scanning microscopy (CLSM) system, employing NIS-Elements software (Nikon), was used for acquiring two-dimensional confocal images.

The same plating procedure was followed for samples analyzed by flow cytometry. After 24 and 72 h from incubation with 100 μg/mL of Vybrant DiO-labeled L-TAFe NPs in complete DMEM without phenol red (Sigma-Aldrich) at 37 °C, 5% CO_2_, fibroblasts were collected and centrifuged at 2600 rpm for 6 min. The pellet was resuspended and transferred in Cytoflex Tubes (Bio-Rad). FITC events (λ_ex_, 488 nm; λ_em_, 525–540 nm) were acquired using flow cytometry (Cytoflex, Beckman Coulter), setting the measurement threshold to 10^4^ events/sample.

Confocal Raman imaging was also performed to evaluate the internalization of unlabeled L-TAFe NPs. Human primary skin fibroblasts were seeded on Raman-grade calcium fluoride substrates (Crystran, seeding density 2 × 10^4^ cells/cm^2^). The following day, the cells were incubated with 100 μg/mL L-TAFe NPs for 24 and 72 h. The cells were subsequently fixed with 4% paraformaldehyde in PBS for 20 min at 4 °C. Cultures were kept in PBS during spectral acquisition, performed by 60× immersion objective and imaged with a confocal Raman microscope (LabRAM HR Evolution, Horiba). Raman images were constructed based on spectra taken from 1050 different points on an area of 40 × 70 μm^2^. The typical signal of the cells corresponding to phenylalanine residues was followed in the range of 980–1028 cm^–1^; L-TAFe NP signal in the cells was identified by two contributions: Fe–O bonds originating by tannic acid–iron complexes (532–539 cm^–1^), and tannic acid and DSPE-PEG hydrocarbon chain vibrations (1470–1492 cm^–1^). LabSpec 6 software was used to obtain the signal maps; the pixel intensity in the maps is proportional to the integrated peak area.

The antioxidant activity of L-TAFe NPs was evaluated by flow cytometry through the detection of a fluorogenic probe that enhances fluorescence upon ROS production. Human primary skin fibroblasts were seeded in six-well cell culture plates (COSTAR) at a density of 6 × 10^4^ cells/well. After 24 h, they were incubated in complete DMEM without phenol red (Sigma-Aldrich) with different concentrations of L-TAFe NPs (10, 50, 100 μg/mL) and with free tannic acid, l-ascorbic acid, and N-acetyl-l-cysteine (all of the molecular antioxidants were used at a concentration of 5 μM that corresponds to the concentration of tannic acid encapsulated in L-TAFe NPs 10 μg/mL). Analyses were performed after 24 h of incubation at 37 °C, 5% CO_2_, by diluting CellRox Green Reagent (Invitrogen) to a final concentration of 5 μM in complete medium without phenol red. The cells were rinsed once in PBS, then the reagent was added and incubation was performed for 30 min at 37 °C, 5% CO_2_. Successively, cells were trypsinized for 5 min and centrifuged at 2600 rpm for 6 min; after removal of the medium, the pellet was resuspended in 1 mL of PBS and aliquoted into two Cytoflex tubes (Bio-Rad), 500 μL each. One tube was kept as control and the second one was treated with 2.5 mM *tert*-butyl hydroperoxide (TBH, Sigma-Aldrich) as a prooxidant insult. Data were acquired using flow cytometry (Cytoflex, Beckman Coulter), setting the measurement threshold to 10^4^ events/sample and measuring the fluorescence of the cells in the FITC channel (λ_ex_, 488 nm; *λ*_em_, 525–540 nm). Two time points were considered: 30 and 60 min after oxidative stress induction. At least three independent experiments have been carried out. ROS levels measured in each condition were then normalized with respect to the ROS levels of the corresponding control cells (at 30 and 60 min, respectively). The results were, therefore, expressed as “normalized ROS increment” with respect to controls, set to 1 by the normalization, to highlight the increase of ROS levels in each condition.

To assess protective effects from L-TAFe NPs against oxidative stress damage, human primary skin fibroblasts were seeded in 48-well cell culture plates at a density of 1.5 × 10^4^ cells/well. The day after seeding, cells were incubated in complete DMEM without phenol red (Sigma-Aldrich) with 10 μg/mL of L-TAFe NPs. After 24 h, the culture media containing residual particles that were not internalized was removed and replaced with fresh DMEM without phenol red, containing 5 mM of TBH. After 1 h, cell viability was assessed by performing the WST-1 assay, as previously described. The concentration of TBH was chosen as the one giving the highest effect, allowing a meaningful interpretation of differences with respect to cells pretreated with L-TAFe NPs, avoiding exerting, at the same time, a strong impairment of cell viability that would affect data interpretation (Figure S3).

Human primary skin fibroblasts pretreated with 10 μg/mL of L-TAFe NPs for 24 h and then exposed to TBH-induced acute oxidative stress (with the same protocol used for WST-1 assay) were analyzed with the live/dead viability/cytotoxicity assay kit (Invitrogen). In this case, 3 × 10^4^ cells/well were seeded in 24-well black μ-plate Ibidi. After 1 h from the acute stress induction, the cells were washed with PBS and then treated with the kit components following the protocol indicated by the supplier. Briefly, 150 μL of a PBS solution containing 2 μM calcein-acetyoxymethyl (live cell staining), 4 μM ethidium homodimer-1 (dead cell staining), and 1 μg/mL Hoechst 33342 (nuclei staining) was added to each well, and the cells were incubated for 45 min at room temperature. After three washes with PBS, a CLSM system with NIS-Elements software (Nikon) was utilized to acquire two-dimensional confocal images.

### *In Vivo* Tests

Planarians belonging to the species *D. japonica* (fissiparous GI strain)^42^ were maintained in pure water added with 0.8 mM NaHCO_3_, 0.4 mM MgSO_4_, 0.077 mM KCl, and 2.5 mM CaCl_2_. Prior to experimental procedures, planarians were starved for 1 week. Animals were fed three times a week with chicken liver and maintained in a dedicated incubator at 18 °C in the dark. All planarian experiments were performed in compliance with the Italian law and with the European Directive 2010/63/EU.

Before performing toxicological experiments concerning L-TAFe NPs protection against oxidative stress, a preliminary assay to assess the biocompatibility of the nanoparticles at 10 and 100 μg/mL was performed. Both concentrations proved to be safe for planarians after 7 days of incubation (Figure S4). For this reason, a concentration of 50 μg/mL was chosen for further evaluations. For each toxicological experiment, 10 groups of five specimens were each put in a different well of a 12-well plate. Then, 5 mL of planarian water containing L-TAFe NPs at a concentration of 50 μg/mL was added in each planarian-containing well. A matched control plate with 5 mL of plain planarian water *per* well was kept as a control. The plates were incubated overnight, and the following day, TBH was added to the media as a chemical prooxidant insult. From preliminary investigations on TBH toxicity, nine TBH concentrations were selected to test possible antioxidant protective effects of L-TAFe NPs in the 50–450 μM range; such concentrations were each tested on a single well of the two plates. A well without TBH was kept both in the L-TAFe NP-treated and in the control plates. Animals were daily checked for the phenotype dead/alive, up to 7 days from the nanoparticle administration, with the exception of the 3rd day from TBH addition. As a 50 μg/mL L-TAFe NP dispersion was found to be perfectly tolerable for planarians during early trials, a 100% survival prior to TBH treatment was considered an essential prerequisite for an experiment to be carried out to completeness. Five biological replicates were performed so that a total of 25 specimens per experimental class were used. As a positive and non-nanotechnological control, we tested diphenyleneiodonium chloride (DPI, 2.5 μM, Sigma-Aldrich), an antioxidant generally used on planarians.^43^ Representative pictures of live or dead planarians for selected time points and experimental classes were acquired with a Stemi 305 KMAT stereomicroscope (Zeiss) on purposely prepared specimens, i.e., sacrificed in 2% HCl and fixed in 4% formaldehyde.

### Statistical Analysis

The statistical analysis was performed by the one-way ANOVA parametric test with Bonferroni’s mean comparison method using OriginLab Software.

Concerning data analysis of *in vivo* tests, a time-consistent mortality score was assigned to each well. According to this criterion, the earlier a planarian died, the higher the score of its group. We started from an individual score of 8 for animals died on the day of TBH administration, down to 1 for specimens found dead on the last day of observation. An individual score of 0 was assigned to the surviving planarians. The mortality score of a well was computed as the sum of the individual scores of all worms it contained. For each experimental class, we calculated the average mortality score over five experimental replicas and data dispersion as standard error of the mean. Statistical significance was evaluated, when needed, through two-tailed unpaired *t*-tests (α = 0.05).

## Results and Discussion

### Physicochemical Characterization of L-TAFe NPs

**Figure 1:**
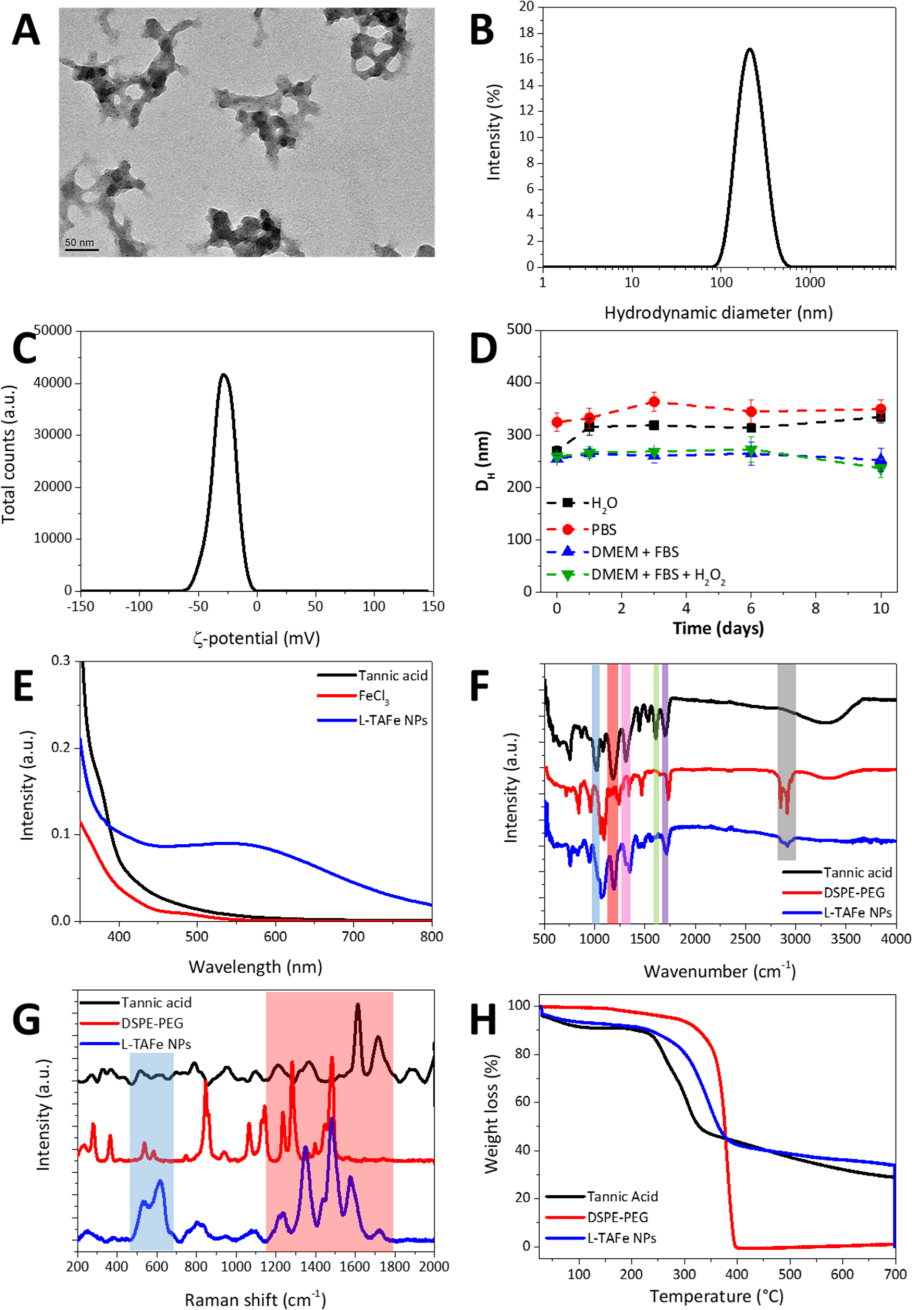
(A) TEM image of L-TAFe NPs. (B) Intensity distribution (%) as a function of the hydrodynamic diameter of L-TAFe NPs. (C) ζ-potential (mV) distribution of L-TAFe NPs. (D) Hydrodynamic diameter *D*_H_ (nm) of L-TAFe NPs in different conditions: water (black squares), PBS (red circles), DMEM + FBS (blue triangles), and DMEM + FBS + H_2_O_2_ (green triangles). (E) UV/vis spectra of tannic acid (black), FeCl_3_ (red), and L-TAFe NPs (blue). (F) FTIR spectra of tannic acid (black), DSPE-PEG (red), and L-TAFe NPs (blue). (G) Raman spectra of tannic acid (black), DSPE-PEG (red), and L-TAFe NPs (blue). (H) Thermograms of tannic acid (black), DSPE-PEG (red), and L-TAFe NPs (blue).

L-TAFe NPs stabilized with DSPE-PEG were synthesized by nanoprecipitation, by modifying a procedure first proposed by Tang et al.^24^ Tannic acid is soluble in water; however, by mixing it with FeCl_3_, hydrophobic tannic acid–Fe^3+^ complexes were readily generated due to the coordination of the metal ion by the organic ligand, forming a three-dimensional network.^21,44,45^ An initial Fe^3+^/tannic acid molar ratio of 3.2 led to the self-assembly of purple complexes.^24^ Nevertheless, without any stabilization, the bare tannic acid–Fe^3+^ complexes tended to quickly aggregate and precipitate. The addition of DSPE-PEG to tannic acid–Fe^3+^ complexes allowed the formation of nanoscale networks, as shown by the TEM image in Figure 1A. L-TAFe NPs have an average size of around 150 nm, with an irregular morphology due to the growth in three dimensions of the coordination complex, similar to that observed by Liu et al.^44^

Dynamic light scattering (Figure 1B) confirms that L-TAFe NPs in water have an average hydrodynamic diameter of 201 ± 4 nm and a polydispersity index (PdI) of 0.093 ± 0.030. Moreover, as evident from the intensity distribution in Figure 1B, L-TAFe NPs presented a fairly monodisperse population despite the irregular morphology. L-TAFe NPs showed a negative ζ-potential equal to −29 ± 1 mV, as also depicted in Figure 1C; this value should guarantee colloidal stability to the nanoparticles, preventing their aggregation and destabilization. In addition, the presence of the PEG chain on the coating should also impart steric stability, making this system suitable for biological applications. The stability over time of L-TAFe NPs has been evaluated in different conditions, i.e., in water, PBS, DMEM + FBS (10%), and DMEM + FBS (10%) + 100 μM H_2_O_2_ (to simulate a biological environment under oxidative stress conditions) (Figures 1D and S5). The hydrodynamic diameter of L-TAFe NPs did not change within 10 days from dilution in the corresponding buffers, suggesting their stability in a wide temporal range, and in different conditions simulating biological media. The smaller average hydrodynamic diameters of L-TAFe NPs in DMEM + FBS (10%) and DMEM + FBS (10%) + 100 μM H_2_O_2_ reported in Figure 1D are due to the presence of proteins that contribute to the hydrodynamic diameter obtained by cumulant analysis. As observable from Figure S5, the intensity distribution in these conditions showed peaks at small diameters (<30 nm). Nevertheless, the main peak attributed to L-TAFe NPs is similar to that observed in water and PBS and does not change over time.

The formation of a coordination complex between tannic acid and iron was also confirmed with UV/vis spectroscopy by the appearance of a charge transfer band with a maximum at around 550 nm (Figure 1E) in L-TAFe NPs. The absence of this band in tannic acid and FeCl_3_ UV/vis spectrum confirms that it was originated by the formation of complexes. For comparison, the color of the sample and the UV/vis spectrum were also assessed in PBS (Figure S6) after diluting the concentrated stock dispersion of L-TAFe NPs in the corresponding buffers. As expected, at basic pH (7.4 in PBS), the color of L-TAFe NPs changes toward a more reddish nuance, with respect to the dark purple color of L-TAFe NPs in MilliQ water (slightly acidic, pH ≈ 5). Contextually, the main absorbance band of L-TAFe NPs in PBS moved to slightly lower wavelengths with respect to the nanoparticles in water. This trend is expected: at pH between 3 and 6, tannic acid and Fe^3+^ form a bis(tannic acid) complex with the metal ion, that can reversibly switch to the tris complex at pH > 6.^24^ However, different to that observed by Tang et al., the transition from the bis- to the tris complex in our L-TAFe NPs was not complete; in fact, at pH 7.4, a much evident change in color and blue shift of the ligand-to-metal charge transfer band (down to 496 nm) is expected. The mild changes observed in our system may be ascribed to a much more kinetically frozen system and a more compact phospholipid coating that does not easily allow the exchange of metal ions with the external environment.

Figure 1F shows the FTIR spectra of tannic acid (black curve), DSPE-PEG (red curve), and L-TAFe NPs (blue curve). The TA spectrum presented the typical peaks of the phenol group at around 1190 cm^–1^ (*ν*_C–O_, red box in Figure 1F), of the aromatic system at 1606 cm^–1^ (*ν*_C=C_, green box), and the ester groups at around 1716 cm^–1^ (*ν*_C=O_, violet box), 1313 cm^–1^ (*ν*_sC–O_, pink box), and 1040 cm^–1^ (*ν*_asC–O_, pink box).^46^ The same contributions were found in the L-TAFe NP spectrum. The DSPE-PEG spectrum showed the peaks of the C–H stretching at around 2860 and 2910 cm^–1^ (gray box); the presence of these peaks also in L-TAFe NPs confirmed the successful coating of the complexes with the lipid.

In Figure 1G, typical Raman spectra of tannic acid (black curve), DSPE-PEG (red curve), and L-TAFe NPs (blue curve) in the region 200–2000 cm^–1^ are reported. In L-TAFe NPs, the appearance of the peaks at around 533 and 617 cm^–1^ (blue box), associated with the chelation of Fe^3+^ by the phenolic oxygen in tannic acid, can be observed;^47^ this contribution is absent in both tannic acid and DSPE-PEG spectra. The peaks in the region between 1100 and 1700 cm^–1^ (red box) are, instead, associated to tannic acid ring vibrations^47^ and to hydrocarbon chain vibrations^45^ of DSPE-PEG; these contributions are both present in the L-TAFe NP spectrum, confirming the presence of the lipid in the complex.

The thermograms of tannic acid, DSPE-PEG, and L-TAFe NPs are shown in Figure 1H. Tannic acid has a very complex thermal degradation pathway (black curve) due to the fact that the different layers of the macromolecule degrade at different stages.^48^ Below 200 °C, a 6% weight loss due to dehydration of tannic acid was observed. As can be seen from Figures 1H and S7A, two main weight loss contributions could be identified at 259 and 304 °C, correlated to the degradation of the outer layer of gallic acid units that correspond to almost 50% of the total tannic acid weight. Above 400 °C, the gallic acid units in the inner layer also started to decompose. However, at 700 °C, there was still almost 29% of the compound that was not completely degraded. This residual mass has been associated to the production of char during thermal degradation of tannic acid under nitrogen, and it is in line with the values reported in the literature.^48^ At 700 °C, in fact, most of the carbon–oxygen-containing functional groups in the glucose central ring and the gallic acid units directly linked to it were reported to be intact; this is due to the condensation and cross-linking of the inner aromatic rings through intermolecular and intramolecular reactions, forming a sort of protection against further degradation for the remaining C–O–C groups until 700 °C, as reported by Xia et al.^48^

Conversely, DSPE-PEG thermal decomposition, reported in Figures 1H and S7A, showed a main weight loss at around 380 °C, and the lipid was completely degraded at 700 °C. In the L-TAFe NP thermogram (Figure 1H and its relative derivative weight curve in Figure S7A), a first 6% weight loss due to water evaporation could be observed below 200 °C. At higher temperatures, the contributions due to both tannic acid and DSPE-PEG could be identified, even if, due to relatively similar degradation temperatures of the two compounds, the derivative weight curve is composed of a single, yet complex, peak between 200 and 400 °C. This thermal event was correlated to a weight loss of about 55% of the total mass of L-TAFe NPs. A tentative deconvolution of this peak is shown in Figure S7B. The first two contributions are associated to tannic acid degradation (red and green curves), while DSPE-PEG degradation can be attributed to the peak at a higher temperature (blue curve). By analyzing the area of these three contributions and correlating it to the total mass of the sample, it can be estimated that DSPE-PEG accounted for 7.6% of the weight of L-TAFe NPs. From ICP, the amount of Fe^3+^ in L-TAFe NPs was estimated to be 5.4 wt %; therefore, assuming the deconvolution is correct, tannic acid should be accounted for the 87 wt % of L-TAFe NPs, representing almost the majority of the nanoparticles. The prevalence of tannic acid in the nanoparticles with respect to the other components can also be inferred by the amount of the residual mass (char) at the end of the heating ramp of L-TAFe NPs (33.9%). In fact, by subtracting the amount of iron (determined by ICP), the organic char formed in L-TAFe NPs should be around 28.5%, very close to the value found for plain tannic acid. Combining the information obtained by ICP and TGA, we could estimate that the Fe^3+^/tannic acid molar ratio in L-TAFe NPs was around 1.9. This is in line with the literature data, where the stoichiometry of Fe^3+^–tannic acid complexes was reported to be 2:1 at pH values between 3 and 7.^49^

Even though the obtained value is in good agreement with the literature, it must be stressed that the estimation of tannic acid amount in L-TAFe NPs might not be accurate due to approximations in the fitting procedures of the TGA data and to the partial overlap between the contribution of tannic acid and DSPE-PEG in the thermal degradation event. Nevertheless, these results suggest that L-TAFe NPs are mainly composed of tannic acid; therefore, they represent a good system to deliver high payloads of antioxidant molecules, different from that obtained with conventional nanosystems.

**Figure 2:**
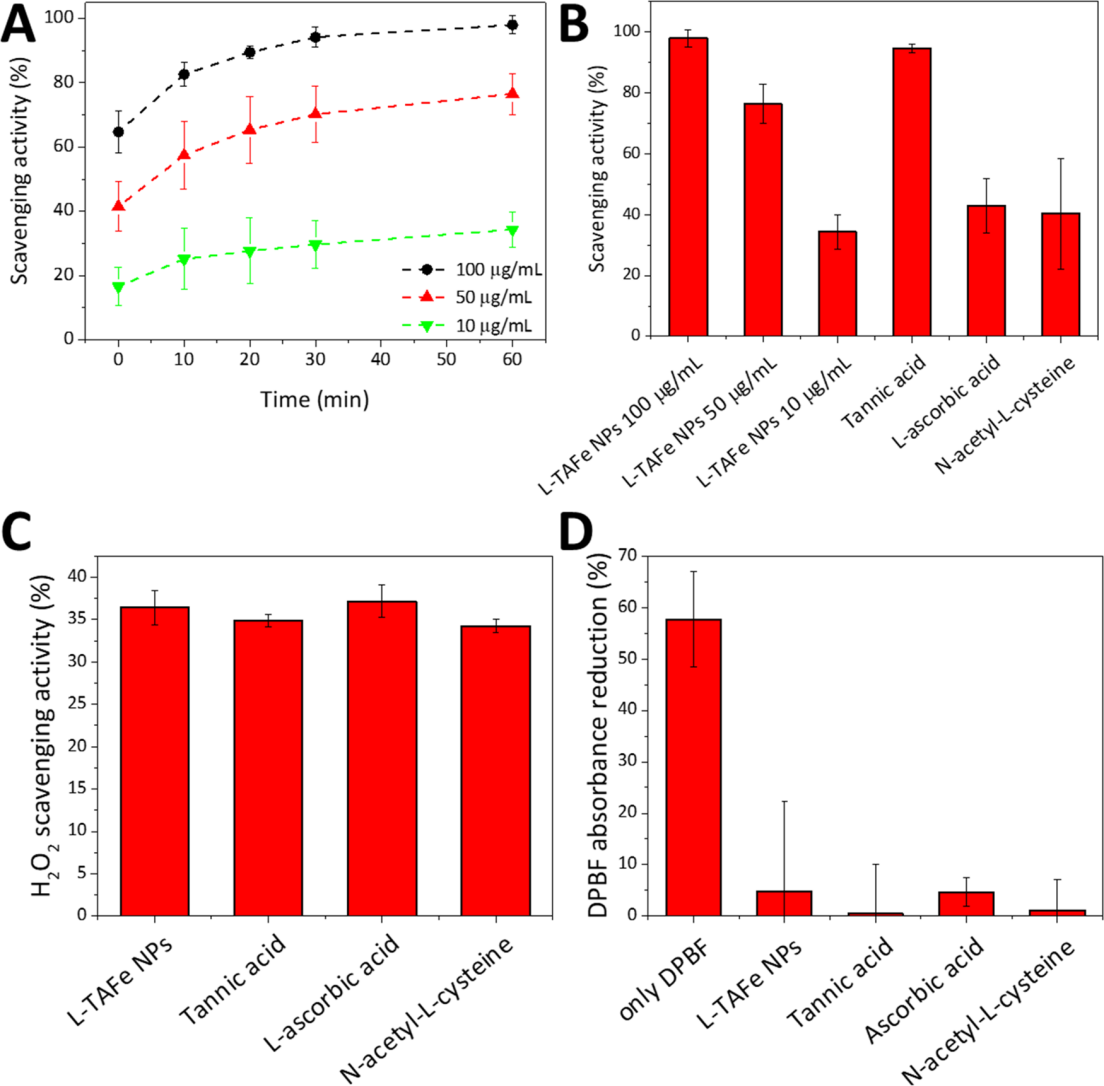
Free radical scavenging activity of different concentrations of L-TAFe NPs (100, 50, 10 μg/mL) evaluated with the DPPH assay at (A) different time points, and (B) at plateau (60 min), compared to free tannic acid, l-ascorbic acid, and N-acetyl-l-cysteine (5 μM, corresponding to the concentration of free tannic acid in 10 μg/mL L-TAFe NPs). (C) H_2_O_2_ scavenging activity (%) of L-TAFe NPs (10 μg/mL), free tannic acid, l-ascorbic acid, and N-acetyl-l-cysteine (all of them 5 μM). (D) DPBF absorbance reduction (%) due to singlet oxygen without antioxidants or with L-TAFe NPs (10 μg/mL), free tannic acid, l-ascorbic acid, and N-acetyl-l-cysteine (all of them 5 μM).

The antioxidant activity of L-TAFe NPs was first evaluated with the TAC assay that measures the ability of an antioxidant molecule to reduce Cu^2+^ to Cu^1+^ as compared to Trolox, a standard antioxidant. The assay showed that a dispersion of 1.2 mg/mL of L-TAFe NPs had an antioxidant capacity corresponding to Trolox 2.2 mM. To better characterize L-TAFe NP antioxidant behavior, their free radical scavenging activity was also evaluated with the DPPH assay.^38^ DPPH is a rather stable free radical with a strong absorption band at 517 nm; in the presence of free radical scavengers or hydrogen donors, DPPH turns into diphenylpicrylhydrazine that does not absorb at 517 nm. Figure 2A,B shows the scavenging activity of three different concentrations of L-TAFe NPs (100, 50, and 10 μg/mL) against a 50 μM alcoholic solution of DPPH. The reduction of the intensity of DPPH absorption band at 517 nm was monitored over time, and the DPPH scavenging activity of L-TAFe NPs (in %) was calculated at each time point. As highlighted in Figure 2A, regardless of the L-TAFe NP concentration, the maximum amount of DPPH that the specific L-TAFe NP concentration could reduce was reached after ≈30 min. At plateau (≈60 min), a dispersion of 100 μg/mL L-TAFe NPs was able to convert 98 ± 3% of DPPH into its reduced form, while the dispersions at 50 and 10 μg/mL could reduce 76 ± 6 and 34 ± 5% of DPPH, respectively (Figure 2B). The DPPH scavenging activity of L-TAFe NPs was compared to that of free tannic acid, l-ascorbic acid, and N-acetyl-l-cysteine at a concentration of 5 μM, which corresponds to the concentration of tannic acid in a L-TAFe NP dispersion of 10 μg/mL. As evident from Figure 2B, both l-ascorbic acid and N-acetyl-l-cysteine had a similar DPPH scavenging activity (43 ± 9 and 40 ± 18%) with respect to 10 μg/mL of L-TAFe NPs, while free tannic acid showed a higher DPPH scavenging activity (94 ± 1%) with respect to all of the other tested antioxidants. On the other hand, when considering the H_2_O_2_ scavenging activity, all of the antioxidants showed a similar behavior, with about 35% of H_2_O_2_ scavenged in the tested conditions. The same behavior could be observed with respect to the singlet oxygen scavenging activity: no difference could be detected among L-TAFe NPs, free tannic acid, l-ascorbic acid, and N-acetyl-l-cysteine (Figure 2D). The singlet oxygen can break the π-system in DPBF, with consequent inability to absorb visible light.^40^ Without any antioxidant agent, a 58 ± 9% reduction of DPBF absorption was observed. However, when L-TAFe NPs, free tannic acid, l-ascorbic acid, or N-acetyl-l-cysteine were incubated with DPBF during singlet oxygen production, the absorption reduction of DPBF was significantly counteracted (5 ± 17% in L-TAFe NPs, 0.5 ± 9% in free tannic acid, 5 ± 3% in l-ascorbic acid, and 1 ± 6% in N-acetyl-l-cysteine), proving that the antioxidants are able to efficiently scavenge singlet oxygen.

The antioxidant activity of free tannic acid has been previously studied. For example, Choi et al. and Gülçin et al. showed that tannic acid displays ROS scavenging properties toward H_2_O_2_, ^•^OH^–^, and ^•^O_2_ and can inhibit lipid peroxidation:^16,50^ this is in agreement with our results. In a recent study, tannic acid was demonstrated to be very efficient in scavenging DPPH radicals, superoxide anion, peroxyl, nitric oxide, peroxynitrite, and to reduce ferric ions. Moreover, thanks to its ability to absorb UV light, tannic acid was able to prevent photodamage by inhibiting lipid peroxidation, depolarization of mitochondrial transmembrane potential, DNA damage, and expression of matrix metalloproteinase-1 protein.^17^ A similar behavior is, thus, expected by L-TAFe NPs, even though the encapsulation in nanoparticles and the interaction with Fe^3+^ might alter the antioxidant properties of L-TAFe NPs with respect to free tannic acid. The results in Figure 2, however, show that the antioxidant abilities in L-TAFe NPs are mostly preserved. The higher activity toward the DPPH radical of tannic acid might be explained with a more effective exposure of the reaction sites in the free molecule with respect to the complexed one, that could make it more readily available to interact with DPPH. Nevertheless, the higher reactivity of tannic acid could be detrimental when interacting with biological systems, both *in vitro* and *in vivo*, as the molecule could be easily degraded. It is also worth noting that free tannic acid is a potent chelator of iron ions and its antioxidant activity has also been linked to this specific function. In fact, tannic acid can form stable complexes with Fe(II) ions, avoiding their involvement in Fenton reactions responsible for ^•^OH formation.^51^ Tannic acid antioxidant activity has also been correlated to its copper chelating ability, and it has been observed that it can form complexes able to entrap ^•^OH radicals.^52^ L-TAFe NPs being an already complexed form of tannic acid, we can speculate that these last features might be less prevalent in the antioxidant activity of L-TAFe NPs. Nevertheless, a detailed study concerning the mechanism of action of L-TAFe NPs is beyond the scope of this work.

### L-TAFe NP Biocompatibility Evaluation in Human Primary Skin Fibroblasts

**Figure 3:**
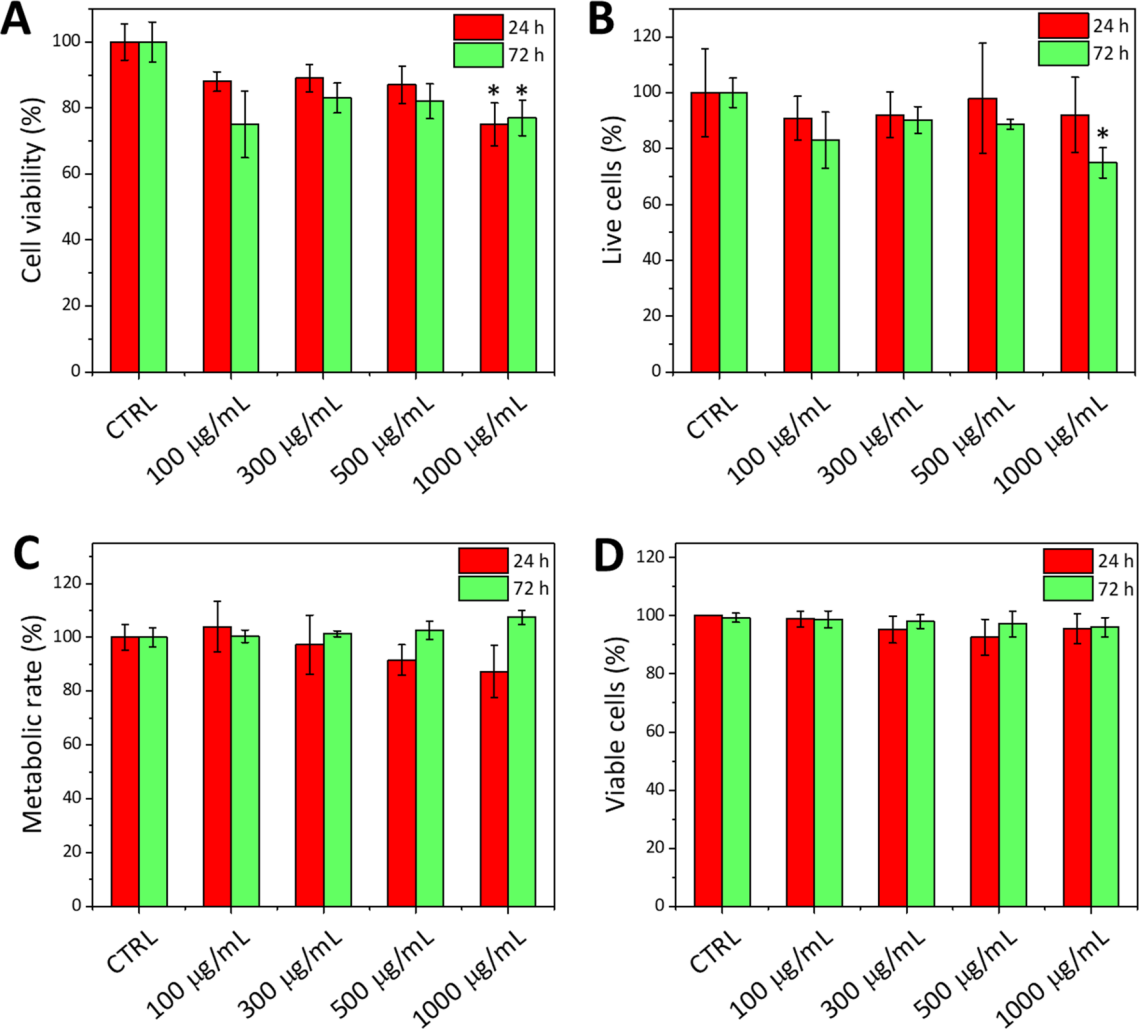
(A) WST-1 assay performed on human primary skin fibroblasts incubated with different concentrations of L-TAFe NPs for 24 h (red) and 72 h (green). (B) PicoGreen assay performed on human primary skin fibroblasts incubated with different concentrations of L-TAFe NPs for 24 h (red) and 72 h (green). Both analyses (A and B) were normalized to control cultures (CTRL; *p < 0.05 with respect to CTRL). (C) Alamar Blue assay performed on human primary skin fibroblasts incubated with different concentrations of L-TAFe NPs for 24 h (red) and 72 h (green). (D) Viable cells (%) after incubation with different concentrations of L-TAFe NPs for 24 h (red) and 72 h (green), determined with the Trypan Blue exclusion assay.

The metabolic activity and the proliferation rate of fibroblasts were analyzed by WST-1 and PicoGreen assays, 24 and 72 h after administration of different concentrations of L-TAFe NPs (Figure 3). Both at 24 and 72 h, no significant effect on the cell metabolism could be detected up to a concentration of 500 μg/mL of L-TAFe NPs; at 1000 μg/mL, a statistically significant (*p* < 0.05) decrease of cell viability to 75 ± 6 and 77 ± 5%, at 24 and 72 h, respectively, was observed (Figure 3A). Regarding the proliferation rates in Figure 3B, a reduction by ≈25% was appreciable at the highest concentration of nanoparticles after 72 h of treatment (1000 μg/mL). These results suggest that L-TAFe NPs are biocompatible in a wide range of concentrations; nevertheless, since a slight impact on cells could be observed at concentrations higher than 500 μg/mL, the following experiments concerning L-TAFe NP internalization and antioxidant activity were performed at concentrations below this threshold (<500 μg/mL). Both Alamar Blue and Trypan Blue exclusion assays (Figure 3C,D) confirmed the biocompatibility of L-TAFe NPs as no statistically significant reduction of the cell metabolic rate or of the number of viable cells could be detected at any L-TAFe NP concentration.

### Evaluation of L-TAFe NP Cellular Internalization in Human Primary Skin Fibroblasts

**Figure 4:**
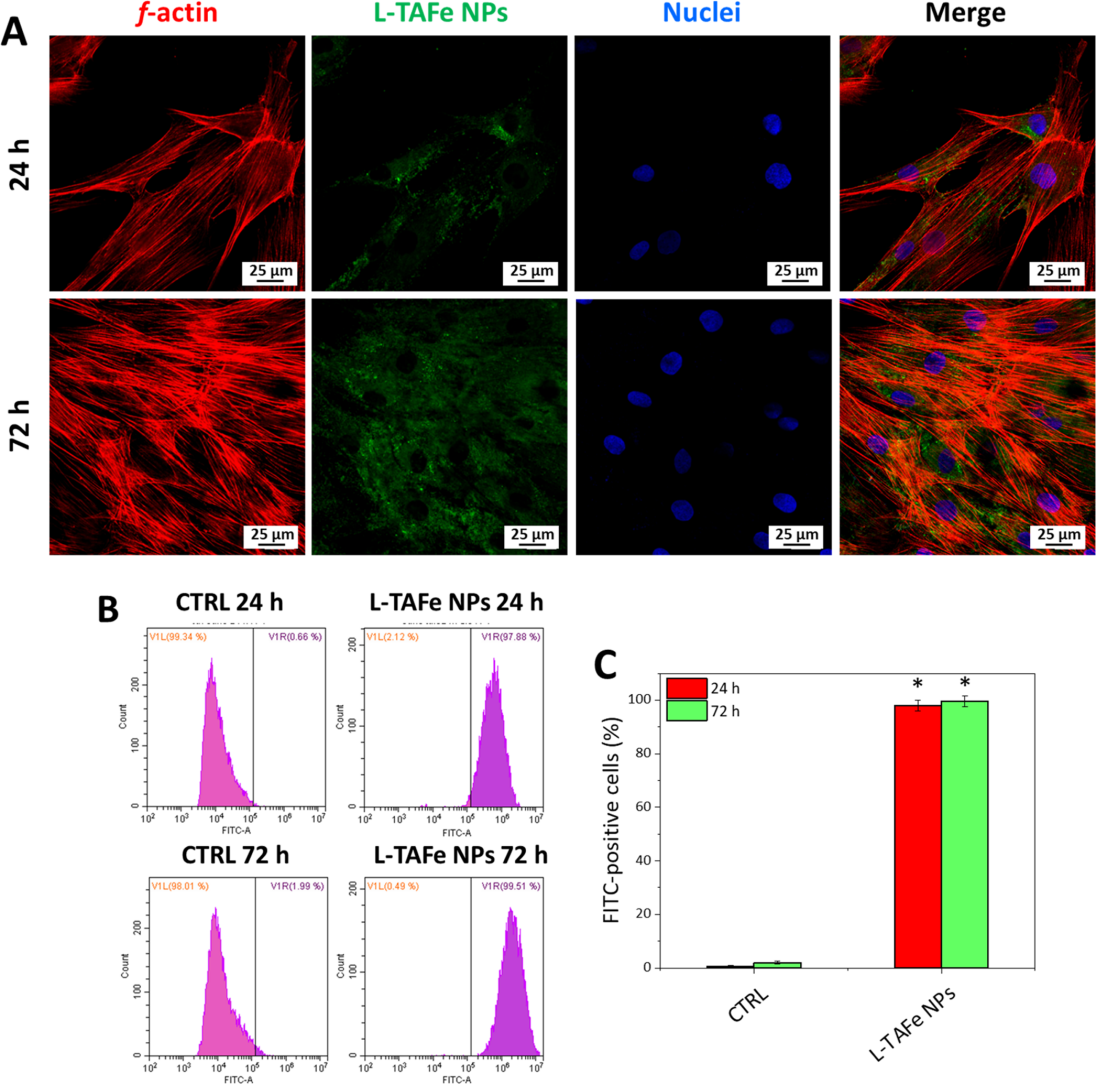
(A) Representative fluorescence confocal images of human primary skin fibroblasts treated with Vybrant DiO-labeled L-TAFe NPs (100 μg/mL, in green) for 24 and 72 h; nuclei (blue) and f-actin (red) were also stained. (B) Representative flow cytometry plots obtained at 24 and 72 h after treatment with Vibrant DiO-labeled L-TAFe NPs (100 μg/mL) and (C) quantification of Vybrant DiO-labeled L-TAFe NPs positive cells (%) derived from flow cytometry (*p < 0.05, with respect to control cultures, CTRL).

Confocal imaging was performed on human primary skin fibroblasts, at 24 and 72 h after administration of Vybrant DiO-labeled L-TAFe NPs, to assess their cellular localization (Figure 4A). Images of the single fluorescent channels, namely, Vybrant DiO-labeled L-TAFe NPs (green), F-actin (red), and nuclei (blue), were acquired. As it can be appreciated, Vybrant DiO-labeled L-TAFe NPs displayed a time-dependent internalization and a diffused cytoplasmic localization at both time points. No colocalization with nuclei was observed. Flow cytometry was exploited for quantifying the amount of internalized Vybrant DiO-labeled L-TAFe NPs by measuring the percentage of FITC-positive cells with respect to the control cells (Figure 4B,C). As can be observed, at 24 h, cultures presented 98 ± 2% FITC-positive cells; this value reached 99.5 ± 2% at 72 h. These results support the observations obtained by confocal microscopy, also suggesting that L-TAFe NPs are prevalently internalized already at 24 h after administration.

**Figure 5:**
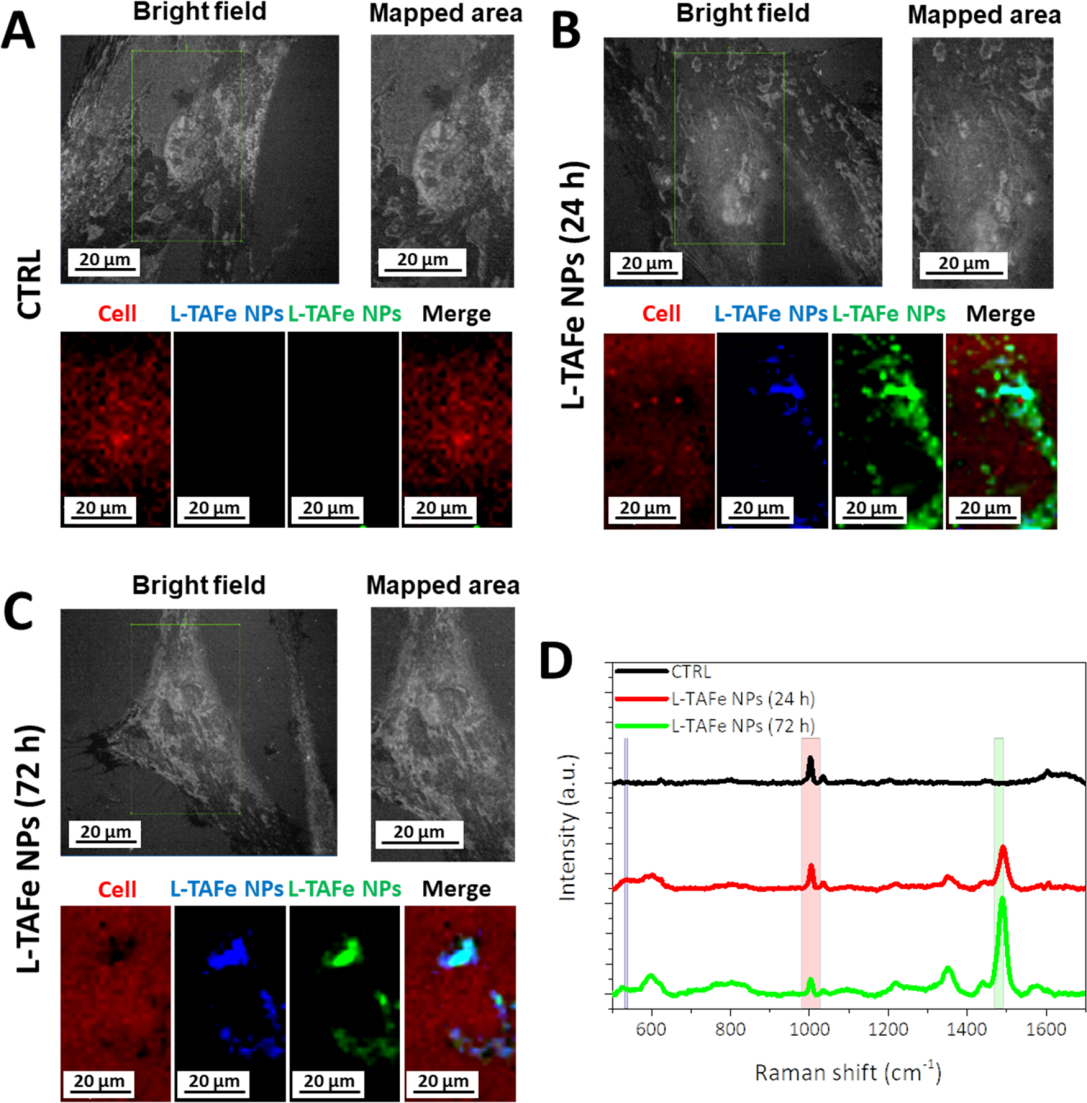
(A–C) Raman microscopy of human primary skin fibroblasts treated with L-TAFe NPs (100 μg/mL) for 24 and 72 h and of control cultures. In the bright field image, the cell area mapped by Raman microscopy is highlighted by the green square and zoomed in the “mapped area”. In this area, the cell signal (red) corresponds to phenylalanine residues (980–1028 cm^–1^), while the L-TAFe NP signal is mapped following two contributions: Fe–O bond in blue (532–539 cm^–1^) and tannic acid and DSPE-PEG hydrocarbon chain vibrations in green (1470–1492 cm^–1^). (D) Representative Raman spectra at a selected point in the map for control cells (CTRL, black) and cells treated with L-TAFe NPs for 24 h (red) and 72 h (green); the colored boxes highlight the characteristic signals of the cells (red box) and L-TAFe NPs (blue and green box).

To confirm these findings with a technique that is not dependent on a labeling procedure, confocal Raman microscopy was performed to follow label-free L-TAFe NP uptake by human primary skin fibroblasts and to map their intracellular localization. Figure 5A–C presents, for each experimental condition (untreated cells and cells treated with L-TAFe NPs at 24 and 72 h), bright field images of the mapped area together with the signals originating from L-TAFe NPs (Fe–O bonds at 533–539 cm^–1^ in blue, and tannic acid and DSPE-PEG hydrocarbon chain vibrations at 1470–1492 cm^–1^ in green) and from cells (phenylalanine residues at 980–1028 cm^–1^ in red), as well as merged images (Merge). It must be noticed that, as evident from Figure 5D, the Raman spectrum of untreated fibroblasts (black curve) does not show any peak that could interfere with L-TAFe NP detection (characteristic signals highlighted by the blue and green boxes in the graph); therefore, the signal appearing at these Raman shifts can unequivocally be attributed to L-TAFe NPs. This is also confirmed by the map of control cells in Figure 5A, where no signal could be detected in the characteristic region of L-TAFe NPs. On the other hand, when fibroblasts were treated with L-TAFe NPs, contributions due to the nanoparticles could be easily detected both at 24 and 72 h, with a good colocalization with the cell signal. From the images, it can be inferred that L-TAFe NPs were diffused in the cell cytoplasm with a perinuclear organization, confirming observations carried out by confocal microscopy.

### *In Vitro* Antioxidant Activity of L-TAFe NPs in Human Primary Skin Fibroblasts

**Figure 6:**
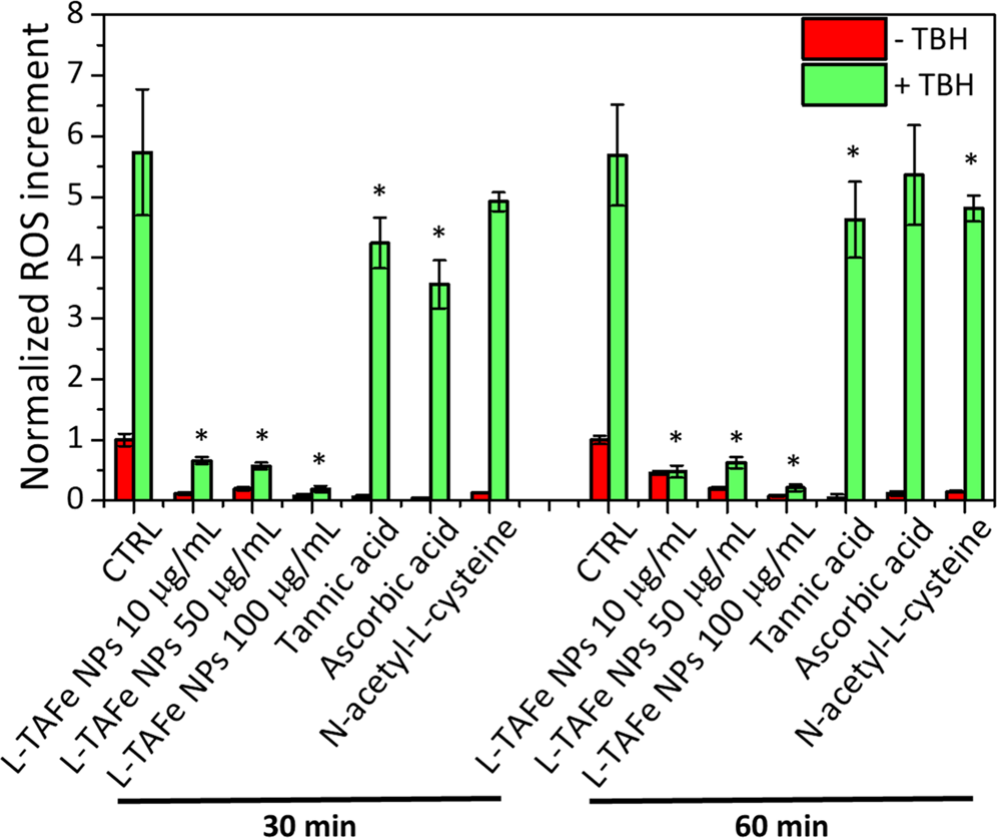
Flow cytometry quantification of CellRox Green in human primary skin fibroblasts treated with different concentrations of L-TAFe NPs and with free tannic acid, l-ascorbic acid, and N-acetyl-l-cysteine (all at 5 μM, corresponding to tannic acid concentration in 10 μg/mL L-TAFe NPs) for 24 h, acquired 30 and 60 min after induction of oxidative stress by TBH (green). The same nontreated experimental classes are reported in red. The ROS increment in each experimental class is normalized to the respective negative control (CTRL) at 30 and 60 min (*p < 0.05, referred to CTRL treated with TBH).

The antioxidant activity of L-TAFe NPs was evaluated upon incubation of human primary skin fibroblasts with increasing amounts of nanomaterials (10, 50, and 100 μg/mL). Flow cytometry was performed 24 h after treatment, staining the samples with CellRox Green Reagent. FITC fluorescence intensity, corresponding to ROS levels, was recorded at 30 and 60 min after oxidative stress induction by TBH. To better highlight the effects of TBH and the protection of L-TAFe NPs, ROS levels of all experimental classes were normalized by those detected in control cells (see Materials and Methods). As can be observed in Figure 6, ROS levels in fibroblasts treated with L-TAFe NPs and analyzed after 30 min were clearly lower with respect to the control cells: for 10, 50, and 100 μg/mL of L-TAFe NPs, ROS levels were, respectively, (0.12 ± 0.02)-, (0.2 ± 0.03)-, and (0.08 ± 0.03)-fold with respect to the control cells (1.00 ± 0.10). A similar behavior was observed 60 min after the treatment (0.46 ± 0.03, 0.20 ± 0.03, and 0.08 ± 0.01 for 10, 50, and 100 μg/mL, respectively). TBH administration induced an evident increase in ROS levels. In fact, in TBH-treated cells at 30 and 60 min, ROS levels were, respectively, (5.73 ± 1.04)- and (5.69 ± 0.83)-fold with respect to the control cells; however, cells pretreated with L-TAFe NPs showed a much lower amount of ROS levels, with values comparable to those observed in cells prior to oxidative stress induction. This result strongly suggests a protective role of L-TAFe NPs at all of the tested concentrations.

The *in vitro* ROS scavenging activity of L-TAFe NPs was compared to that of free tannic acid and the conventional antioxidant molecules, l-ascorbic acid, and N-acetyl-l-cysteine, that were considered also for the preliminary scavenging activity evaluations (Figure 2). The antioxidant compound concentration was set at 5 μM, which corresponded to the estimated concentration of tannic acid encapsulated in 10 μg/mL of L-TAFe NPs, and that was found to be nontoxic for each considered compound (WST-1 results reported in Figure S8). As highlighted in Figure 6, all of the tested molecular antioxidants have a very mild ROS scavenging activity, especially if compared to the effect of 10 μg/mL of L-TAFe NPs. ROS levels in cells pretreated with free tannic acid were (4.24 ± 0.42)- and (4.62 ± 0.61)-fold higher than those in the control cells, respectively, after 30 and 60 min from TBH administration. When cells were pretreated with l-ascorbic acid, ROS were (3.56 ± 0.36)- and (5.36 ± 0.82)-fold higher than those in the control cells, respectively, after 30 and 60 min from TBH administration; while cell pretreatment with N-acetyl-l-cysteine led to ROS levels (4.92 ± 0.15)- and (4.8 ± 0.2)-fold higher than in the control cells, respectively, after 30 and 60 min from TBH administration. In all of these cases (except for N-acetyl-l-cysteine at 30 min and l-ascorbic acid at 60 min), the ROS level reductions with respect to TBH-treated cells without any prior administration of antioxidants is statistically significant (*p < 0.05); nevertheless, at the same concentration, L-TAFe NPs showed a much higher ROS scavenging activity (ROS levels (0.12 ± 0.02)- and (0.46 ± 0.03)-fold at 30 and 60 min, respectively). Interestingly, although the preliminary comparison of the radical scavenging activity of L-TAFe NPs with the molecular antioxidants showed no differences or, in the case of free tannic acid, even a lower efficacy of the NPs, in *in vitro* experiments, the benefits of using L-TAFe NPs is clearly highlighted. Antioxidants in their molecular form are easily degraded and are scarcely internalized by cells; for this reason, their protective action cannot be entirely carried out *in vitro* or *in vivo*.^2^ On the other hand, the encapsulation of tannic acid in L-TAFe NPs not only guarantees a better cellular uptake but also preserves tannic acid antioxidant properties in complex systems.

**Figure 7:**
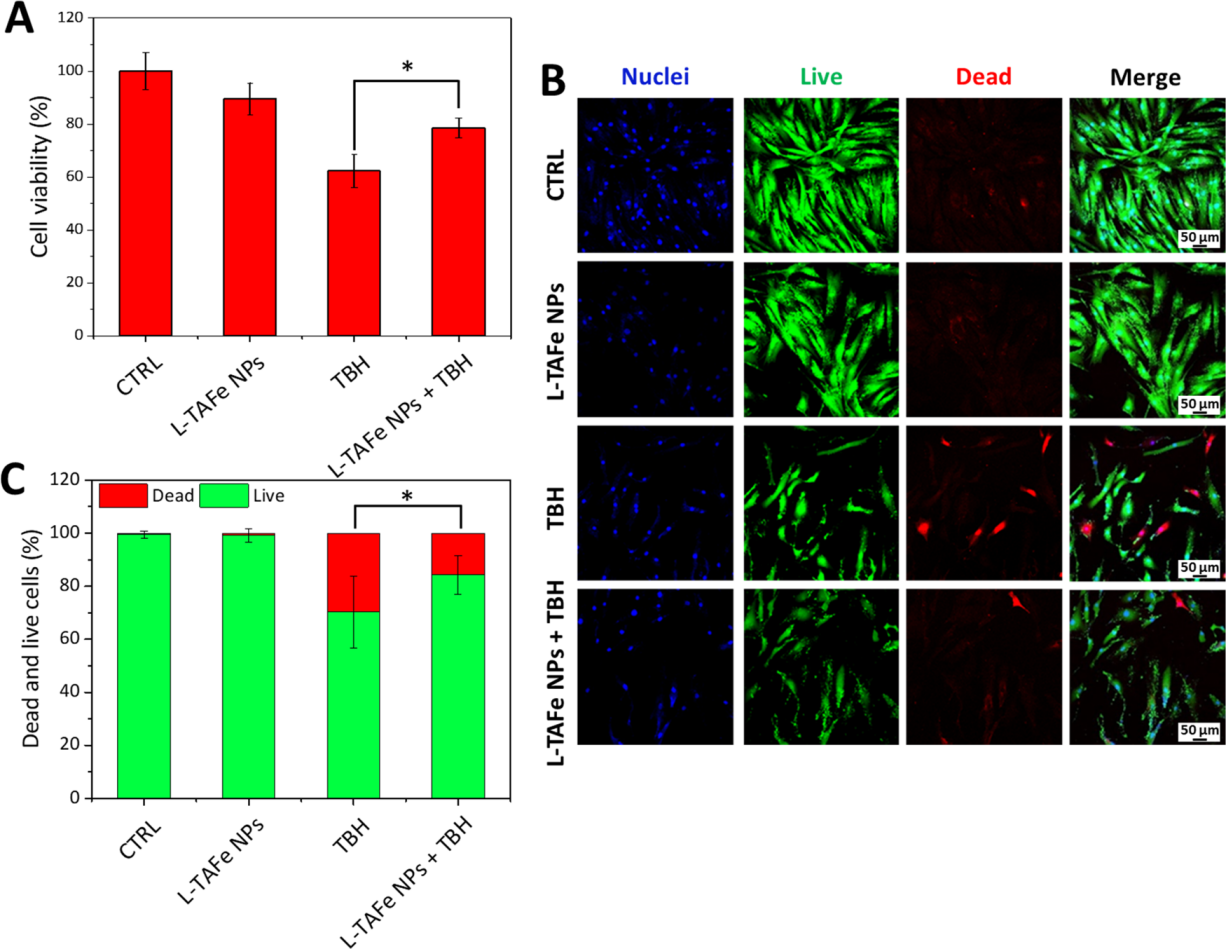
(A) WST-1 assay performed on human primary skin fibroblasts incubated with L-TAFe NPs (10 μg/mL) for 24 h and then treated with TBH (5 mM) to induce oxidative stress. Analyses were normalized to control cultures (CTRL; *p < 0.05). (B) Representative fluorescence confocal images of the live/dead viability assay on human primary skin fibroblasts incubated with L-TAFe NPs (10 μg/mL) for 24 h and then treated with TBH (2.5 mM) to induce oxidative stress. (C) Dead and live cells (%) quantified from the image analysis (*p < 0.05).

To evaluate the protective effect of L-TAFe NPs against acute oxidative stress in human primary skin fibroblasts, the metabolic activity of cells treated with 5 mM TBH for 1 h was evaluated by the WST-1 assay, with or without pretreatment with 10 μg/mL L-TAFe NPs (Figure 7A). Fibroblasts treated with TBH displayed a reduction in the cell viability to 62 ± 6%; however, in cells pretreated with L-TAFe NPs, the oxidative stress only induced a cell viability reduction to 79 ± 4% with respect to the control cells, demonstrating a protective effect of L-TAFe NPs against ROS-induced damage.

Since oxidative stress is often related to strong cytotoxicity, the effect of L-TAFe NPs on TBH-induced damage was also evaluated by the live/dead assay (Figure 7B,C). In the cells treated with an acute TBH insult, the number of dead cells detected was 30 ± 13%, whereas in the samples pretreated with L-TAFe NPs and then exposed to acute oxidative stress, the number of dead cells was reduced to 16 ± 7%: L-TAFe NPs efficiently exerted a protective effect against the damage caused by acute oxidative stress *in vitro*.

### *In Vivo* Protective Effect of L-TAFe NPs against Oxidative Stress in Planarians

**Figure 8:**
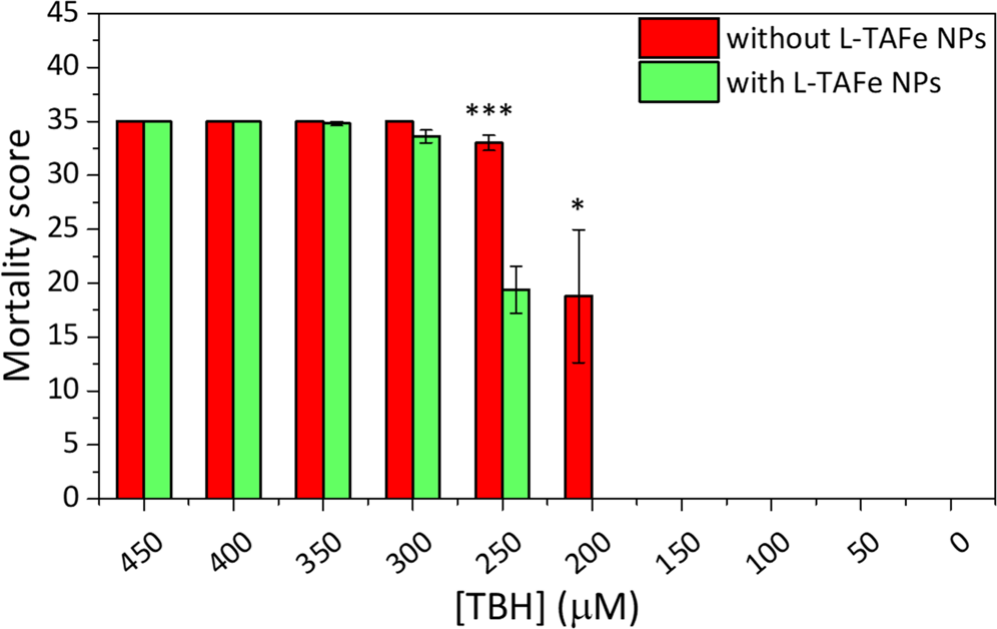
*In vivo* toxicology results. L-TAFe NPs (50 μg/mL) significantly counteract the toxic effect elicited by TBH at concentrations of 250 and 200 μM. Animal viability is completely preserved at 200 μM TBH concentration after L-TAFe NP administration with respect to untreated animals (two-tailed unpaired *t*-test, α = 0.05; *p < 0.05; ***p < 0.001). Plots have been obtained through the assignment of a time-consistent mortality score, starting from 8 for the day of TBH administration down to a score of 1 for day 7 (0 was assigned to survived specimens), followed by averaging scores for each experimental class and computing of standard error.

To confirm the findings observed *in vitro*, the antioxidant properties and the related protective action of L-TAFe NPs against oxidative stress were also tested in an *in vivo* model. Planarian worms are ideal for toxicological studies due to their anatomical and physiological features^36^ and, in particular, *D. japonica* represents an ideal model for high-throughput toxicological assays. In this work, planarians treated with L-TAFe NPs (50 μg/mL) were subjected to oxidative stress by imparting a pro-oxidant chemical insult, TBH, at different concentrations. Animals were daily scrutinized for the macroscopic phenotype dead/alive over several days, and an overall mortality score was calculated on five experimental replicas. As shown in Figures 8 and S9, treatment with L-TAFe NPs was effective in reducing the TBH-elicited mortality on planarians at 250 μM and completely protected animals from death at 200 μM TBH concentration with respect to their control groups, consistent with a radical scavenging effect and the antioxidant activity of L-TAFe NPs. As positive and non-nanotechnological control, we tested diphenyleneiodonium chloride (DPI, 2.5 μM, Sigma-Aldrich), previously used as an antioxidant on planarians.^43^ DPI is an electron transport inhibitor and exerts its antioxidant activity by interfering with nitric oxide synthase, mitochondrial complex I, and cytochrome P-450.^53^ For comparison, at tested conditions, DPI only displayed a trend toward protection against TBH at 150 or 200 μM, but this tendency was not supported by statistical analysis (Figure S10).

## Conclusions

Summarizing the data reported in this work, tannic acid–Fe^3+^ complexes can be easily formulated into functional nanoaggregates, thanks to the stabilizing action of a biocompatible PEGylated phospholipid. The final L-TAFe NPs are stable in several conditions, including those resembling typical biological environments and allow the encapsulation of high payloads of tannic acid, themselves being mainly composed of antioxidant molecules. We characterized the antioxidant activity of L-TAFe NPs *in vitro* and *in vivo*. They demonstrated to be biocompatible and, importantly, displayed significant preventive and antioxidant activity upon oxidative stress induction. These observations lay the foundations for future development of this system as possible antioxidant nanovectors for efficient therapy of oxidative stress-related diseases and offer a new application for tannic acid–Fe^3+^ complexes. In this perspective, further work will be performed for making these nanovectors able to target specific cells of interest.

## Supporting Information Available

Ten supplementary images depicting the chemical formula of tannic acid; normalized absorbance at 570 nm for different concentrations of L-TAFe NPs obtained in the TAC assay (in the linearity range); human primary skin fibroblast viability assay after treatment with different concentrations of TBH for 1 h; *in vivo* biocompatibility results; intensity distribution (%) as a function of the hydrodynamic diameter (nm) of L-TAFe NPs in different conditions; UV/vis spectra of L-TAFe NPs in water and PBS; derivative weight loss (%) of tannic acid, DSPE-PEG, and L-TAFe NPs, and peak deconvolution of L-TAFe NP thermal event; human primary skin fibroblast viability after treatment with 5 μM tannic acid, l-ascorbic acid, and/or N-acetyl-l-cysteine for 24 h; representative micrographs of planarians treated with L-TAFE NPs and/or TBH; and *in vivo* toxicology following DPI treatment (PDF)
