# Supplementary material for: Tannic Acid–Iron Complex-Based Nanoparticles as a Novel Tool against Oxidative Stress
Source: ACS Appl Mater Interfaces. 2022 Mar 30;14(14):15927–41. doi: 10.1021/acsami.1c24576 (PMC9011352; doi:10.1021/acsami.1c24576)
Supplement: Supplementary file 1 — am1c24576_si_001.pdf [file am1c24576_si_001.pdf]

Supporting information

Tannic acid – iron complex-based nanoparticles as a novel tool against oxidative stress

Carlotta Pucci,[,‡,1] Chiara Martinelli,[*,‡,1,†] Daniele De Pasquale,[‡,1] Matteo Battaglini,[1] Nicoletta di Leo,[1,2] Andrea Degl'Innocenti,[1,†] Melike Belenli Gümüş,[1,2] Filippo Drago,[3] Gianni Ciofani[*,1]*

1 Istituto Italiano di Tecnologia, Smart Bio-Interfaces, Viale Rinaldo Piaggio 34, 56025 Pontedera, Italy

2 Scuola Superiore Sant'Anna, The Biorobotics Institute, Viale Rinaldo Piaggio 34, 56025 Pontedera, Italy

3 Istituto Italiano di Tecnologia, Electron Microscopy Facility, Via Morego 30, 16163 Genova, Italy

*Carlotta Pucci: carlotta.pucci@iit.it; *Chiara Martinelli: chiara.martinelli@polimi.it, *Gianni Ciofani: gianni.ciofani@iit.it

‡These authors contributed equally

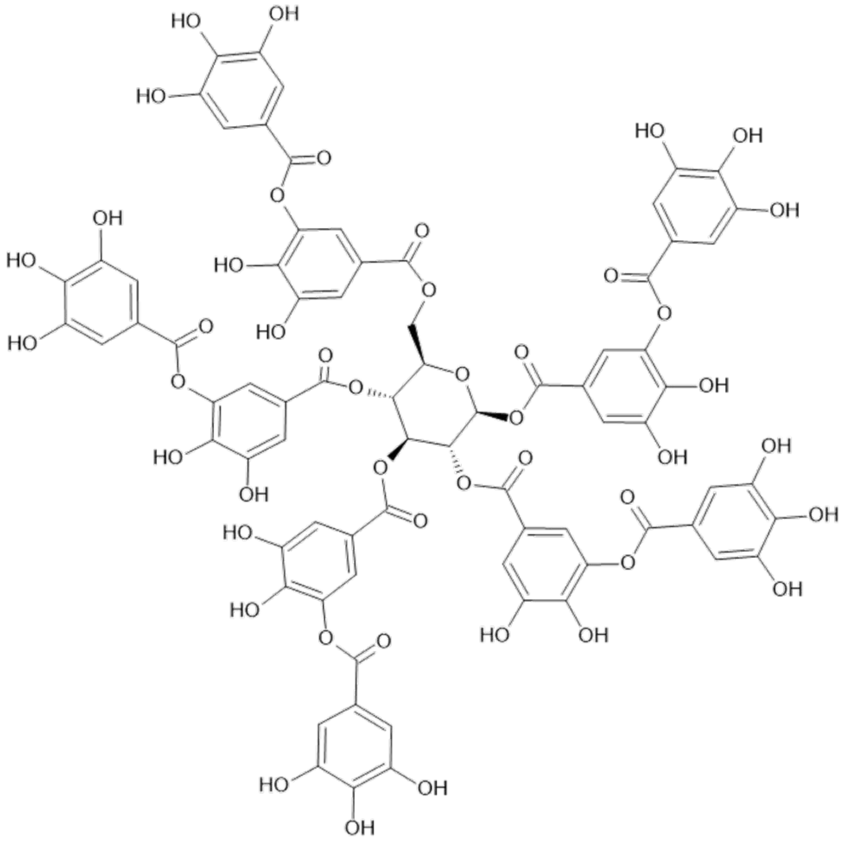

Figure S1. Chemical structure of tannic acid.

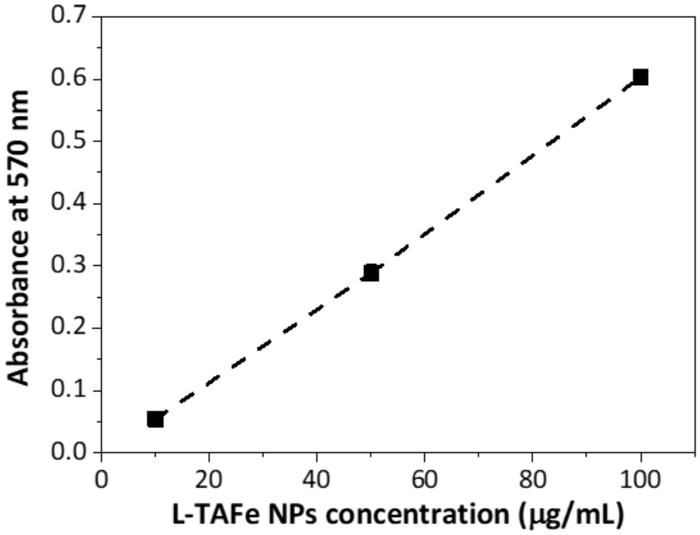

Figure S2. Normalized absorbance at 570 nm for different concentrations of L-TAFe NPs obtained in the total antioxidant capacity assay. The three chosen concentrations fall within the linearity range.

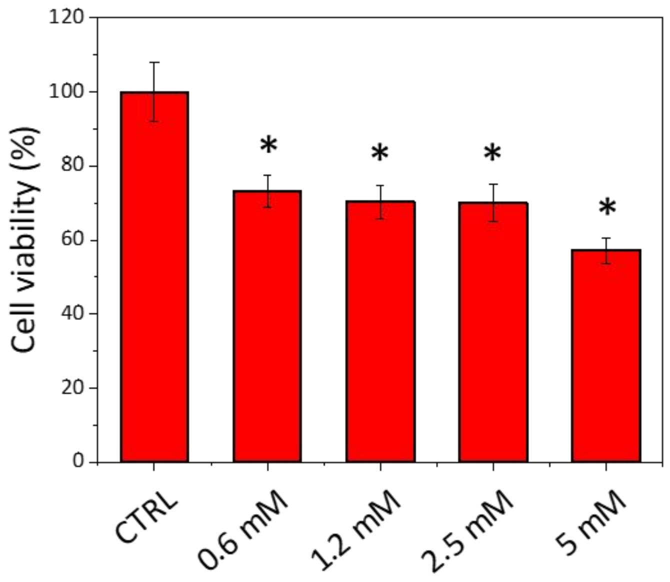

Figure S3. Human primary skin fibroblasts viability after treatment with different concentrations of TBH for 1 h. Analyses were normalized to control non-treated cells (CTRL; * $p < 0.05$ with respect to CTRL).

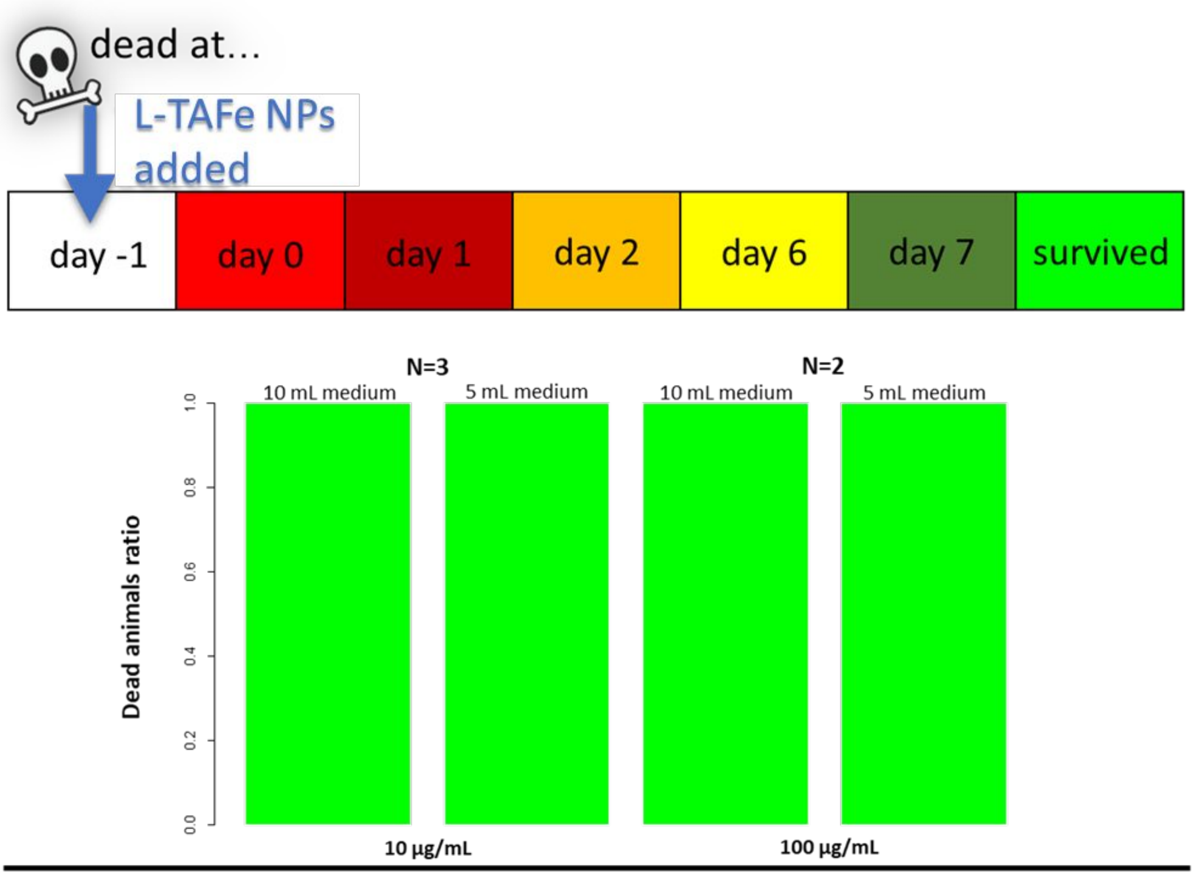

Figure S4. *In vivo* biocompatibility results. Planarians survival is 100% after the administration of L-TAFe NPs at both 10 and 100 µg/mL after seven days of incubation.

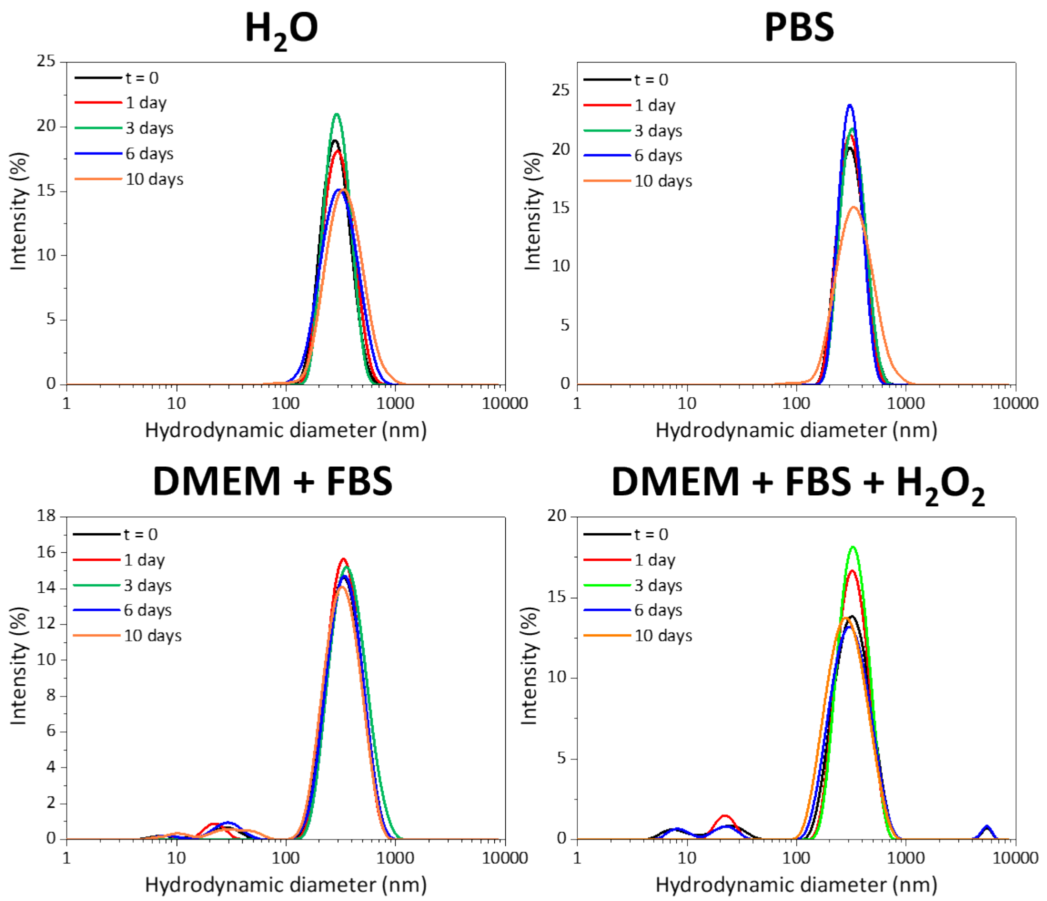

Figure S5. Intensity distribution (%) as a function of the hydrodynamic diameter (nm) of L-TAFe NPs in different conditions (H_2O, PBS, DMEM+FBS 10%, DMEM+FBS+H_2O_2) at different time points (0, 1, 3, 6, 10 days).

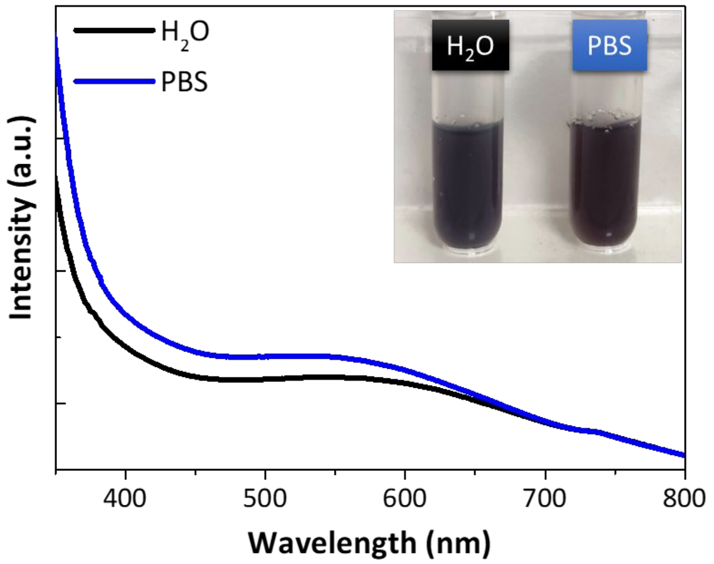

Figure S6. UV/Vis spectra of L-TAFe NPs in water (black) and PBS (blue). In the inset, a picture showing the color of L-TAFe NPs in the two different solutions is reported.

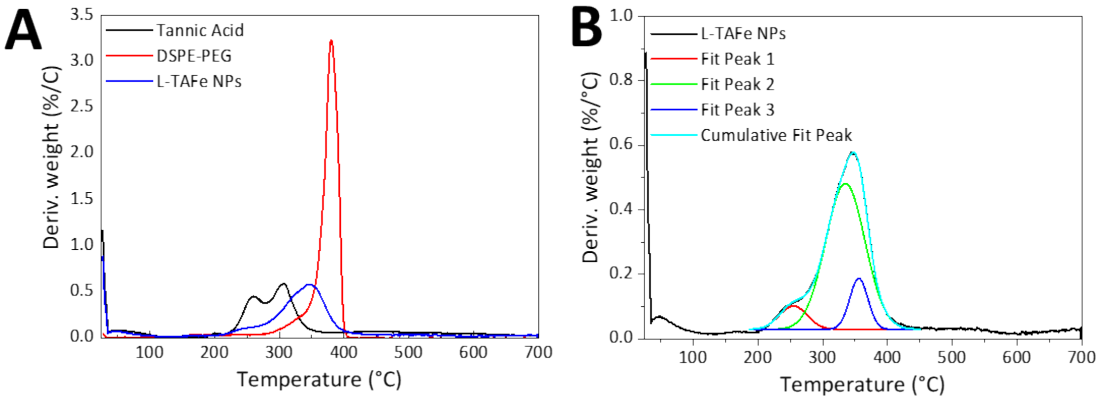

Figure S7. (A) Derivative weight (%) of tannic acid, DSPE-PEG, and L-TAFe NPs. (B) Peak deconvolution of the L-TAFe NP thermal event.

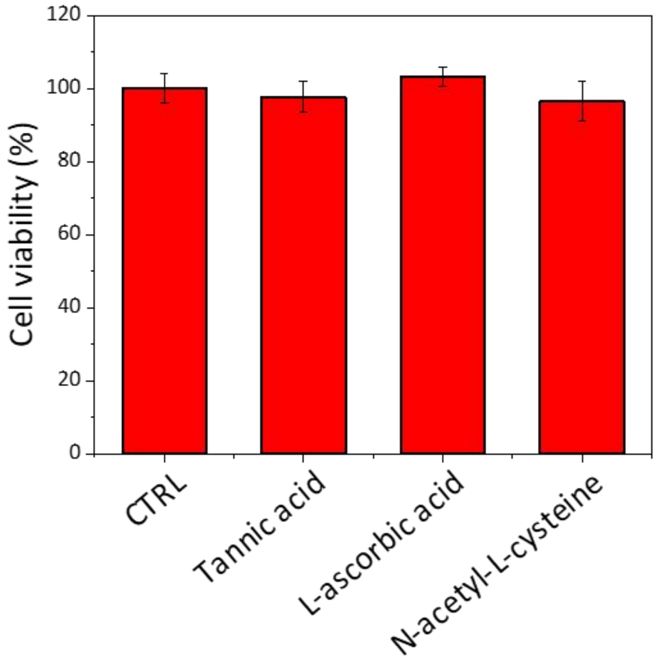

Figure S8. Human primary skin fibroblasts viability after treatment with 5 µM of tannic acid, L-ascorbic acid, or and N-acetyl-L-cysteine for 24 h (WST-1 assay). Analyses were normalized to control non-treated cells (CTRL).

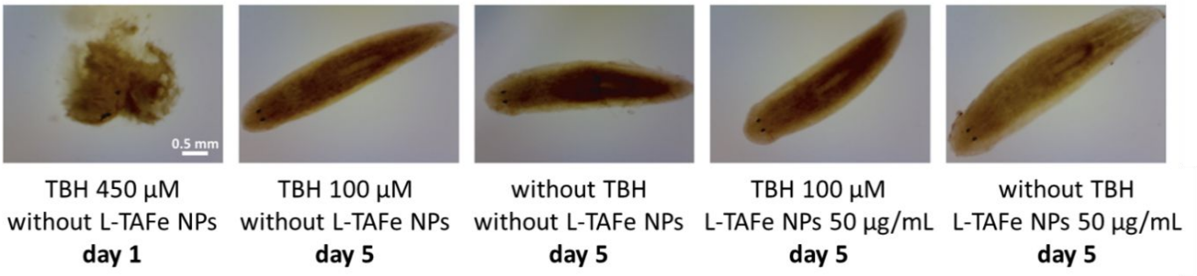

Figure S9. Micrographs showing either dead or live planarians (prepared and fixed for imaging), as typically obtained for each of the indicated conditions in terms of TBH concentration, presence/absence of L-TAFe NPs, and time point (in terms of experimental day). In particular, a freshly dead specimen is shown on the left, and four whole planarians are portrayed in the remaining images. All pictures have the same magnification.

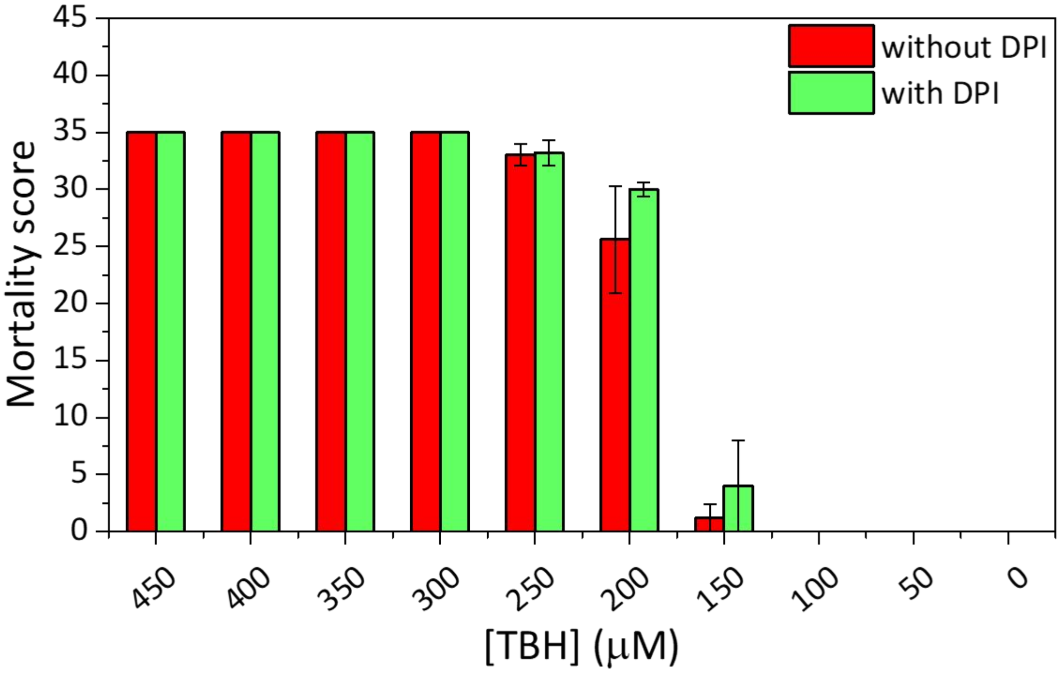

Figure S10. *In vivo* toxicology results following DPI (2.5 µM) administration, used as antioxidant positive control. DPI may exert a modest protective activity at low TBH concentrations (150 µM and 200 µM) compared to untreated animals, but such effects are not statistically significant (two-tailed unpaired *t*-test, α = 0.05). Plots have been obtained through the assignment of a time-consistent mortality score, starting from 8 for the day of TBH administration down to a score of 1 for day 7 (0 was assigned to survived specimens), followed by averaging scores for each experimental class and computing of standard error.
